# Ionic Liquid Directed Spinning of Cellulose Aerogel Fibers with Superb Toughness for Weaved Thermal Insulation and Transient Impact Protection

**DOI:** 10.1021/acsnano.3c05894

**Published:** 2023-09-12

**Authors:** Zhongsheng Liu, Zhizhi Sheng, Yaqian Bao, Qingqing Cheng, Pei-Xi Wang, Zengwei Liu, Xuetong Zhang

**Affiliations:** † Suzhou Institute of Nano-tech and Nano-bionics, Chinese Academy of Sciences, Suzhou 215123, P. R. China; ‡ Division of Surgery & Interventional Science, University College London, London NW3 2PF, U.K.

**Keywords:** nanoporous aerogel fiber, highly oriented structure, strength, toughness, hydrogen bond

## Abstract

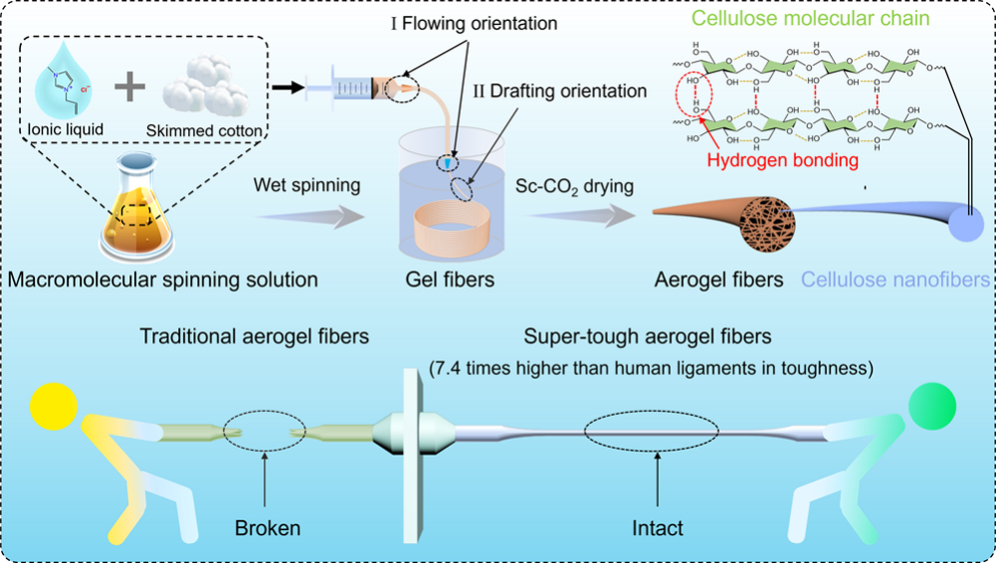

Aerogel fibers, combining the nanoporous characteristics of aerogels with the slenderness of fibers, have emerged as a rising star in nanoscale materials science. However, endowing nanoporous aerogel fibers with good strength and high toughness remains elusive due to their high porosity and fragile mechanics. To address this challenge, this paper reports supertough aerogel fibers (SAFs) initially started from ionic-liquid-dissociated cellulose via wet-spinning and supercritical drying in sequence. The supertough nanoporous aerogel fibers assembled with cellulose nanofibers exhibit a high specific surface area (372 m^2^/g), good mechanical strength (30 MPa), and large elongation (107%). Benefiting from their high strength and elongation, the resultant cellulose nanoporous aerogel fibers show ultrahigh toughness up to 21.85 MJ/m^3^, much outperforming the known aerogel materials in the literature. Moreover, the toughness of this nanoporous aerogel fiber is 7.4 times higher than that of human knee ligaments, and its specific toughness is comparable to that of commonly used solid polyester fibers. In addition, we also verified the weavability, desirable thermal insulation performance, and supertoughness to resist the transient impact of SAFs. The long-sought strategy to simultaneously resolve the strength and toughness of nanoporous aerogel fibers, in combination with the biodegradable nature of the cellulose, provides multifaceted opportunities for broad potential applications, including lightweight wearable textiles and beyond.

Aerogel fibers are a prototypical class of materials that have recently received increasing attention due to their fascinating properties, such as high porosity, low thermal conductivity, and low density. These features have promised nanoporous aerogel fibers for a wide range of applications in fields such as thermal insulation,^1−6^ wearable fabrics,^7−10^ water capture,^11^ electromagnetic shielding,^12^ heat transfer devices,^11^ artificial muscles,^13,14^ and so on.^15−19^ For example, to conquer the difficulties of silica bulk materials, silica aerogel fibers with tunable transparency are fabricated with good flexibility that can be bent to 360° and recovered to the initial state.^18^ The hygroscopic holey graphene aerogel fibers possess the flexibility to be bent and woven into wearable fabrics for water capture and microwave protection.^11^ Polyimide aerogel fibers prepared via the sol–gel confining transformation method show desirable mechanical and thermal insulation properties with fire retardance.^1^ Taking advantage of the aerogel nanoconfinement effect, the introduction of guest liquids into the aerogel fibers has broadened their ultimate mechanics. For instance, the polyethylene glycol (PEG) in between the graphene sheets has led to the strength enhancement of PEG/graphene aerogel fibers.^20^ Infusing Kevlar aerogel fibers with a phase change material such as paraffin, the obtained nanocomposite structure achieves a satisfactory tensile strength of 30 MPa. Moreover, relying on the bending stiffness design, paraffin/Kevlar aerogel fibers with elaborate diameter control can be produced into shape memory artificial muscles upon temperature regulation.^15^ Despite their significant potential, one of the primary challenges nanoporous aerogel fibers face is how to individually, let alone simultaneously, enhance their strength and toughness due to their high porosity and fragile mechanics.

High porosity has given nanoporous aerogel fibers instinctively weak mechanical performance. However, few studies suggest that enhancing the orientation of the aerogel nanoscale building blocks can improve the mechanical properties of aerogel materials.^21,22^ Through directional densification and carbonization, the laminated aramid nanofiber/carbon nanotube hybrid aerogel film with preferential building block orientation shows an abrupt increase in both the mechanical strength and electrical conductivity.^23^ In addition, the mechanical properties of cellulose bulk aerogels with high anisotropy by directional freeze-drying are significantly enhanced in lieu of cellulose aerogels with randomly distributed pores.^21^ Recently, Kevlar liquid crystal aerogel fibers with nanoscale building block orientation obtained by liquid crystalline spinning have achieved high mechanical strength as well as desired thermal insulation.^24^ Therefore, the nanostructural orientation arrangement in nanoporous aerogel fibers may be an effective way to get better mechanical properties of aerogel fibers, especially for their toughness.

In this work, we introduce supertough aerogel fibers (SAFs) initially started from cellulose macromolecules based on an ionic-liquid-assisted spinning technique. Cellulose spinning solution formed by dissolving cellulose into an ionic liquid has exhibited high stability and ensured strong mechanical strength after gelation. The mechanical strength of the final cellulose nanoporous aerogel fiber can be effectively improved by the construction of highly oriented nanofibers and the enhancement of cross-linking points among these nanofibers. The high toughness of the cellulose nanoporous aerogel fibers is related to the deformation of the three-dimensional aerogel nanoporous network, the stretchable cellulose macromolecular chains, and the intermolecular and intramolecular hydrogen bonds formed among the hydroxyl groups on the macromolecular chain. The combined increase in mechanical strength and elongation of the cellulose nanoporous aerogel fibers makes it possible to achieve ultrahigh toughness (up to 21.85 MJ/m^3^). To the best of our knowledge, this is the maximum tensile toughness for nanoporous aerogel fibers among the values reported in the literature. It should be noted that the elongation of this nanoporous aerogel fiber is also the highest among the reported aerogel materials. Benefiting from good mechanical strength and high toughness, these SAFs also show good weaveability, and the resulting weaved product has shown satisfactory thermal insulation performance as well as desirable transient impact protection. This work guides the design and preparation of advanced nanoporous aerogel fibers with enhanced toughness for applications in wearable devices, lightweight thermal insulation, and other emerging fields.

## Results and Discussion

**Scheme 1:**
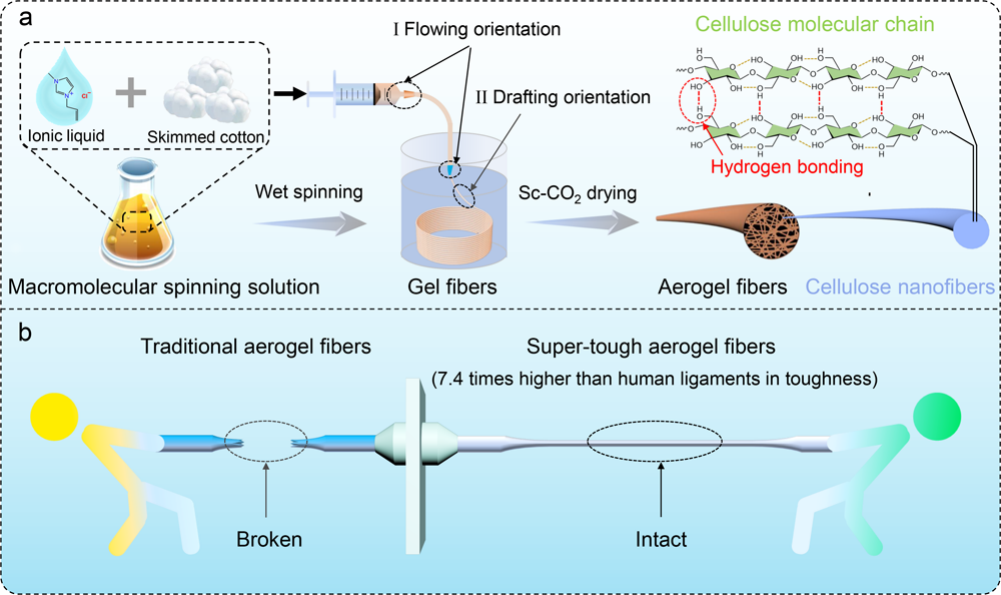
(a) Schematic Fabrication Process of the Supertough Nanoporous Cellulose Aerogel Fibers with a Hierarchical Structure. (b) Schematic Performance Comparison between Traditional Nanoporous Aerogel Fibers and Supertough Nanoporous Aerogel Fibers

In order to achieve nanoporous aerogel fibers with desirable mechanical properties and high toughness, a fabrication strategy based on ionic-liquid-assisted spinning technology is proposed in Scheme 1a, which mainly involves the dissolution of cellulose with ionic liquid as the spinning dope, wet-spinning, solvent exchange, and supercritical CO_2_ drying processes in sequence. Given the widespread distribution and diverse sources (wood, cotton, etc.) of cellulose in nature, it is employed as a raw material in the production of nanoporous aerogel fibers. It is noteworthy that cotton, with a cellulose content approaching 100%, serves as a readily available source of cellulose when defatted (Figure S1a). Currently, most cellulose aerogels or hydrogels are formed by directly cross-linking and entangling preformed nanofibers via hydrogen bonding to create a three-dimensional network structure.^25−27^ Herein cellulose was initially dissolved into specific ionic liquids, such as 1-allyl-3-methylimidazole chloride, with the state of the macromolecular solution rather than nanofiber dispersion.^28,29^ Next, wet-spinning was applied to extrude the cellulose solution into a coagulation bath, which was composed of ethanol for gelling to form gel fibers. During the spinning process, cellulose spinning solution is extruded from a syringe into a certain length of tube and then exits from a nozzle into the coagulation bath, inducing sufficient interval of flow orientation. Meanwhile, drafting orientation is achieved for cellulose gel fibers by drawing the fibers using a rotating disc. After complete gelation, the gel fibers were transferred to ethanol for solvent exchange. Subsequently, supercritical CO_2_ drying was applied to obtain the final cellulose nanoporous aerogel fibers with a hierarchical structure, which are formed through the self-assembly of cellulose macromolecular chains into nanofibers, followed by the three-dimensional network formation of the gel fibers through hydrogen bonding interactions among the nanofibers during the gelling process. It is well-known that the cellulose skeleton is composed of cellulose macromolecules, which are linear chains of ringed glucose molecules with repeating units of two anhydroglucose rings bridged via C–O–C covalent bonds. Abundant hydroxyl groups in the cellulose macromolecules enable the formation of both intrachain and interchain hydrogen bonds (Scheme 1a), which can undergo breaking and reforming events under tension. Compared with traditional nanoporous aerogel fibers with low mechanical strength and poor elongation, supertough cellulose nanoporous aerogel fibers with a hierarchical structure can withstand higher tensile force without fracture (Scheme 1b).

**Figure 1:**
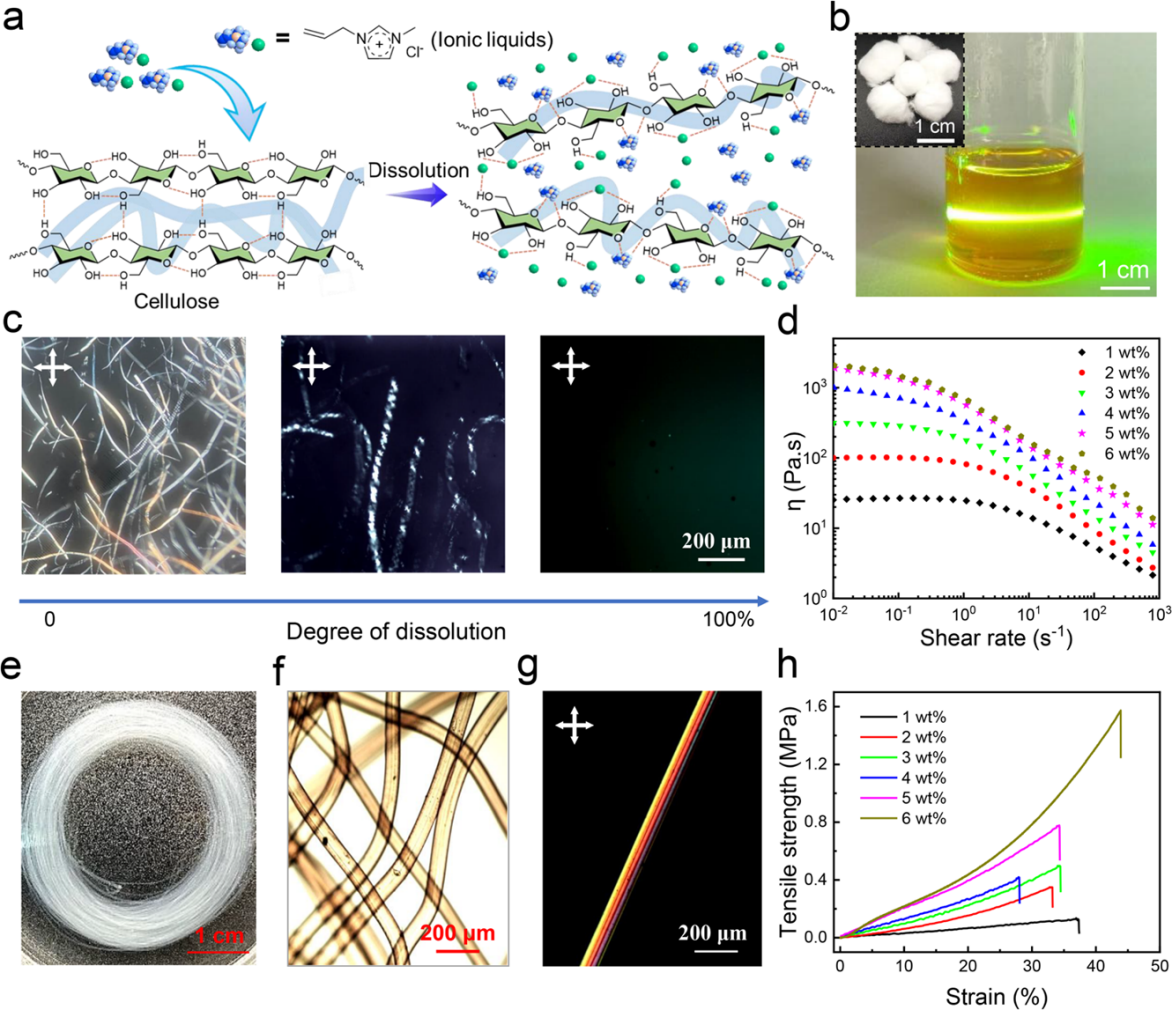
Dissolution mechanisms of cellulose and characterization of cellulose gel fibers. (a) Schematic diagram of the dissolution mechanism of cellulose in ionic liquids. (b) Photograph of cellulose solution with the Tyndall effect. The inset shows a photograph of defatted cotton. (c) Images of cellulose under the polarized light of the microscope at various dissolution degrees. (d) Log–log plots of apparent viscosity as a function of shear rate for different mass fractions of cellulose solutions. (e) Optical picture of cellulose hydrogel fibers. (f,g) Image of cellulose hydrogel fibers under normal light (f) and polarizing light microscope (g). (h) Stress–strain curves of cellulose hydrogel fibers with different mass fractions (1–6 wt %).

Ionic liquids, typically green solvents, have possessed a superior capacity to dissolve cellulose.^30^ The dissolution mechanism of cellulose in ionic liquids [AMIM]^+^Cl^–^ is the result of the combined action of anions and cations.^28^ The ion pairs in the ionic liquid dissociate to individual Cl^–^ and AMIM^+^ ions, where Cl^–^ ions associate with hydroxyl proton in the cellulose, while the free cations AMIM^+^ complex with the cellulose hydroxyl oxygen, breaking the hydrogen bonds in cellulose and hence resulting in the dissolution of cellulose (Figure 1a).^28^Figure 1b demonstrates that the cellulose spinning solution prepared using ionic liquids has a Tyndall effect, indicating that the diameter of its molecular chain entanglement reaches the size of colloid particles.^31^ As the cellulose content in the spinning solution increases, its color also gradually darkens (Figure S1b). In addition, the dissolution process of cellulose in ionic liquids was also observed by a polarized light microscope. At the beginning of dissolution, cotton fibers with a random orientation are clearly visible. After being dissolved for some time, the undissolved cotton fibers are obviously reduced and discontinuous. Upon the complete dissolution, the microscope field will be completely darkened without any visible fibers (Figure 1c). Once the defatted cotton (cellulose) is completely dissolved, the cellulose macromolecules are well-dissolved in the ionic liquid. The nuclear magnetic resonance (NMR) spectra of the spinning solution indicate that the characteristic peaks of the ionic liquid are clearly observed, while the signals of cellulose are hardly seen due to the low cellulose concentration (Figure S2). Rheological studies have shown that the viscosity of the spinning solution also increases with the increase of cellulose mass fractions (Figure 1d). For example, when the mass fraction of the spinning solution increases from 1 to 6 wt %, the viscosity (the shear rate at 0.01 s^–1^) dramatically increases from 25.3 to 2068.6 Pa·s. Figure S3 shows the infrared (IR) spectra of cellulose solids, ionic liquids, and cellulose solutions with different mass fractions (1–6 wt %), respectively. The position of the characteristic peaks of the cellulose solutions does not change significantly with increasing cellulose content in the solutions due to low concentration of cellulose in the spinning solution. In addition, we investigated the microstructures of the ionic liquid and the spinning solution using transmission electron microscopy (TEM). The results, as shown in Figure S4a,b, did not exhibit typical cellulose nanofibers. Furthermore, energy-dispersive spectroscopy (EDS) spectra (Figure S4c) confirmed the presence of oxygen (O) elements in Figure S4b. Therefore, Figure S4b reveals both the ionic liquid and cellulose, suggesting that the spinning solution composed of dissolved cellulose in the ionic liquid is a macromolecular-level solution rather than a nanofiber dispersion.

After wet-spinning, cellulose gel fibers with various cellulose contents can be obtained. Considering the rapid volatilization of ethanol and its potential impact on the characterization of the gel fibers, the obtained gel fibers were immersed in water to obtain the corresponding hydrogel fibers for further characterization (Figure 1e). Figure 1f,g shows the optical morphology of cellulose hydrogel fibers under normal light (Figure 1f) and polarized light (Figure 1g), respectively. The diameter of cellulose hydrogel fibers is extremely uniform, and the semicrystalline nature of cellulose results in a strong birefringence phenomenon that is clearly observed under polarized light. Since cellulose is a semicrystalline polymer with linear macromolecular chains, when the macromolecular chains are aggregated, they tend to assemble into nanofibers first (as observed in the following SEM image of the resulting nanoporous aerogel fibers), and then, the nanofibers and free macromolecular chains are further aggregated and assembled into a three-dimensional network-like gel. Even after stretching, it retains a better birefringence phenomenon (Figure S5). It is apparent that the *in situ* self-assembly of cellulose macromolecular chains into nanofibers during gelling and the resulting nanofibers’ hydrogen bonding with free cellulose macromolecular chains provide more potential hydroxyl sites for hydrogen bond cross-linking compared to the hydrogen bonding formed by hydroxyl groups on preformed nanofiber surfaces. This increased potential for hydrogen bond cross-linking facilitates the formation of more cross-linking points.^32^ During the spinning process, the orientation of the cellulose macromolecules is increased by the sequential arrangement induced by the shear forces. Furthermore, during the gelation process, the orientation of cellulose nanoscale building blocks in gel fibers is further induced by drawing. The realization of hydrogel fiber nanoscale building blocks with high orientation, which can be further confirmed by the following WAXS patterns of the resulting nanoporous aerogel fibers, is a solid foundation for the final nanoporous aerogel fibers with both high strength and superb toughness. The obtained hydrogel fibers exhibit a gradual decrease in water content as the cellulose mass fraction increases from 1 to 6 wt % (Figure S6). With an increase in the content of cellulose, the tensile strength of the hydrogel fiber drastically increases, with the maximum strength up to 1.57 MPa and the maximum strain up to 44.0% (Figure 1h).

To determine the suitable drying process, hydrogel fibers with the same preparation parameters (initial diameter of ∼249 μm, mass fraction of 5 wt %) were subjected to atmospheric pressure drying, freeze-drying, and supercritical drying,^33−35^ respectively. Figure S7 presents the surface (top) and cross-sectional (bottom) morphologies of cellulose nanoporous aerogel fibers that are obtained by different drying methodologies. It is obvious that the shrinkage of aerogel fibers by atmospheric drying and freeze-drying is significant (atmospheric drying: 127.8 μm, shrinkage rate: ∼48.7%; freeze-drying: 158.5 μm, shrinkage rate: ∼36.3%), while the shrinkage of aerogel fibers obtained after supercritical drying is negligible (242.6 μm) with a shrinkage rate of only ∼2.6%. The drying process aims to replace the liquid solvents within the gel with gases while maintaining the integrity of the gel network structure. However, due to the nanoscale dimensions of the internal pores in the gel, great capillary forces caused by liquid surface tension can occur during the drying process, easily leading to shrinkage of the gel matrix and even collapse of the gel structure. This is likely the reason for the relatively large shrinkage of the aerogel fibers obtained by atmospheric drying and frozen drying. However, for supercritical drying, the solvent in the supercritical state has almost no surface tension, thus allowing the gel to maintain its intact network structure after drying. In addition, the specific surface areas of the aerogel fibers dried by these three approaches are 4.78 m^2^/g (atmospheric pressure drying), 175.21 m^2^/g (freeze-drying), and 371.94 m^2^/g (supercritical drying), respectively (Figure S8). In addition, the fibers after atmospheric pressure drying or freeze-drying show higher mechanical strength than fibers after supercritical drying, attributed to the larger degree of fiber shrinkage and the reduced cross-sectional area (Figures S9 and S10). Considering the structural integrity and acceptable mechanical performance of nanoporous aerogel fibers, the supercritical drying approach is selected for further study.

**Figure 2:**
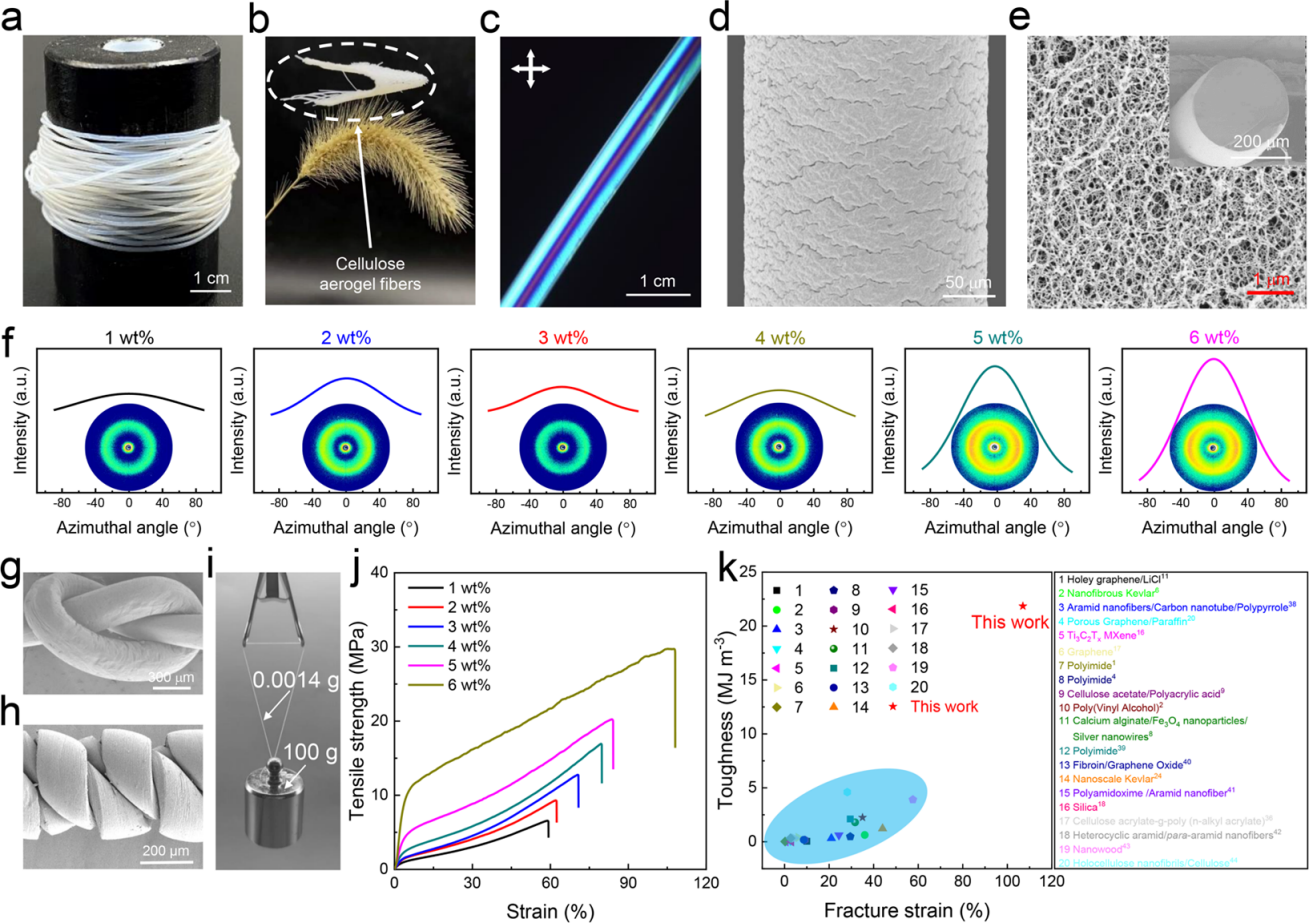
Characterization and properties of SAFs. (a) Optical picture of cellulose aerogel fibers obtained by supercritical drying. (b) Photographs of lightweight aerogel fibers placed on top of green bristle grass. (c) Image of cellulose aerogel fibers under the polarized light of the microscope. (d) Surface SEM images of the cellulose aerogel fibers. (e) Cross-sectional SEM images of the cellulose aerogel fibers. The inset is a low-magnification fiber cross-sectional SEM image. (f) Azimuthal scan wide-angle X-ray scattering (WAXS) patterns (for a rotating anode X-ray source) showing the full width at half-maximum (fwhm) of cellulose aerogel fibers with different mass fractions (1–6 wt %). (g) Surface SEM images of knotted aerogel fibers. (h) Surface SEM images of twisted double cellulose aerogel fibers. (i) Photographs of an aerogel fiber bent into a triangular shape withstanding a load of 100 g. (j) Stress–strain curves of the cellulose aerogel fibers with different mass fractions (1–6 wt %). (k) Comparison of fracture strain and toughness between cellulose aerogel fibers and porous aerogel materials (fibers or films) that have been reported elsewhere.

After supercritical drying, nanoporous cellulose aerogel fibers with different mass fractions (1–6 wt %) can be obtained. With the increase of the initial concentration, the specific surface area of nanoporous cellulose aerogel fibers roughly increased from 264 to 371 m^2^/g, and the average pore diameter was in the range 15–30 nm (Figure S11). The density of cellulose aerogel fibers increases with increasing initial concentration (0.042 to 0.235 g/cm^3^), but their corresponding porosity decreases from 97 to 87% (Figure S12). The obtained nanoporous aerogel fibers can be prepared in the laboratory to be tens of meters in length, exhibiting potential for industrial production (Figure 2a). Moreover, they are lightweight, with densities as low as ∼0.042 g/cm^3^ at a mass fraction of 1 wt %, and a large piece of aerogel fiber textile can be easily placed on top of the Setaria viridis (Figure 2b). It should be noted that the supercritical dried cellulose nanoporous aerogel fibers inherit the birefringence phenomenon (Figure 2c). Figure 2d,e shows the surface (Figure 2d) and cross-sectional (Figure 2e) SEM images of supercritical dried cellulose aerogel fibers, respectively, manifesting that the fiber surface is smooth and the internal mesopores and cellulose nanofibers are uniformly distributed. In order to further investigate the effect of cellulose content on the nanofiber orientation in aerogel fibers, the cellulose nanoporous aerogel fibers with a variety of mass fractions were subjected to wide-angle X-ray scattering tests. Results show that the nanofiber orientation increases with increasing cellulose content in the range of 1–5 wt % (Figure 2f). The Herman’s orientation factor (*f*) was calculated to be 0.2728 (1 wt %), 0.2896 (2 wt %), 0.3403 (3 wt %), 0.3519 (4 wt %), 0.3717 (5 wt %), and 0.3711 (6 wt %) using eqs S2 and S3, respectively.

In addition, the cellulose nanoporous aerogel fibers are very flexible, and single strands can be twisted without any cracks (Figure S13). Moreover, the mechanical strength of two-twisted nanoporous aerogel fibers can be significantly improved compared to a single raw fiber (Figure S14). Even after being knotted or twisted with multiple strands, the nanoporous aerogel fiber does not show any cracks in the SEM image (Figure 2g,h). Notably, an aerogel fiber bent into a triangular shape (0.0014 g) can withstand the weight of an object (100 g) that is ∼71 430 times heavier than its weight (Figure 2i). In order to investigate whether the crystalline structure of cellulose changes before and after dissolution and regeneration, X-ray diffraction (XRD) characterization was carried out on cellulose samples before and after regeneration (Figure S15). It can be seen that the diffraction peaks of cellulose undergo prominent changes after dissolution in ionic liquids and regeneration in ethanol. This can be attributed to the transformation of the native cellulose crystal I structure to the regenerated cellulose crystal II structure.^36^ Thermogravimetric (TG) analysis was conducted to characterize the thermal stability of the cellulose nanoporous aerogel fibers. The results show that the decomposition temperature of the cellulose nanoporous aerogel fibers is slightly lower than that of the raw material, indicating slightly inferior thermal stability. However, the residual mass of the cellulose nanoporous aerogel fibers is higher than that of the raw cotton (Figure S16). This is because the cellulose crystal II form is more prone to conformational flipping and dehydration/decarboxylation, leading to the formation of more residues and therefore higher residual mass.^37^ Additionally, the cellulose nanoporous aerogel fibers are highly hydrophilic due to the abundance of hydroxyl functional groups on their surface. To enable the hydrophobicity, methoxytrimethylsilane was chemically anchored on the surface of the fibers via the cold plasma technique,^24^ achieving a water contact angle of (135 ± 2)° (Figure S17).

In practical applications, good mechanical properties of nanoporous aerogel fibers with both high strength and proper toughness are essential, especially in weaving, where stretching, bending, or folding are often required. Typical stress–strain curves for nanoporous aerogel fibers with different cellulose mass fractions are shown in Figure 2j. Results illustrate that both the tensile strength and strain of nanoporous aerogel fibers increase substantially with increasing cellulose mass fraction, obtaining a maximum strength of 30 MPa at the strain of 107%. The improvement in the mechanical properties of the fibers is attributed to the increase in nanofiber orientation, coincident with the azimuthal scan of WAXS patterns (Figure 2f). Noteworthily, our cellulose nanoporous aerogel fibers can achieve toughness up to 21.85 MJ/m^3^ and tensile strain up to 107%, much outperforming the reported aerogel materials in the literature (fibers or films, Figure 2k and Table S1).^1,2,4,6,8,9,11,16−18,20,24,36,38−44^ For instance, traditional silica aerogel fibers have a low toughness of only ∼0.003 MJ/m^3^ and a fracture strain of ∼3.05%.^18^ In contrast, cellulose aerogel fibers exhibit ∼7280 times higher toughness than that of silica aerogel fibers as well as ∼36 times higher fracture strain. Even compared with high-performance cellulose/silica composite aerogel films (toughness of 3.91 MJ/m^3^ and fracture strain of ∼57%), the cellulose aerogel fibers still exhibit a toughness that is ∼5.6 times higher and a fracture strain that is approximately doubled.^45^ To the best of our knowledge, the toughness of this nanoporous cellulose aerogel fiber is the highest among aerogel fibers that have been reported to date. Moreover, the toughness of this nanoporous aerogel fiber is 7.4 times higher than that of human knee ligaments (toughness of ∼2.94 MJ/m^3^),^46^ with a specific toughness of up to ∼92.97 MJ m^–3^/(g cm^–3^), which is comparable to that of commonly used solid polyester fibers with specific toughness of ∼109.75 MJ m^–3^/(g cm^–3^).^47^ The supertough nanoporous aerogel fibers provide opportunities for the development of wearable textiles and flexible fiber-based devices as well as advanced flexible materials that can adapt to more processing conditions and more complex working environments in practical applications.

**Figure 3:**
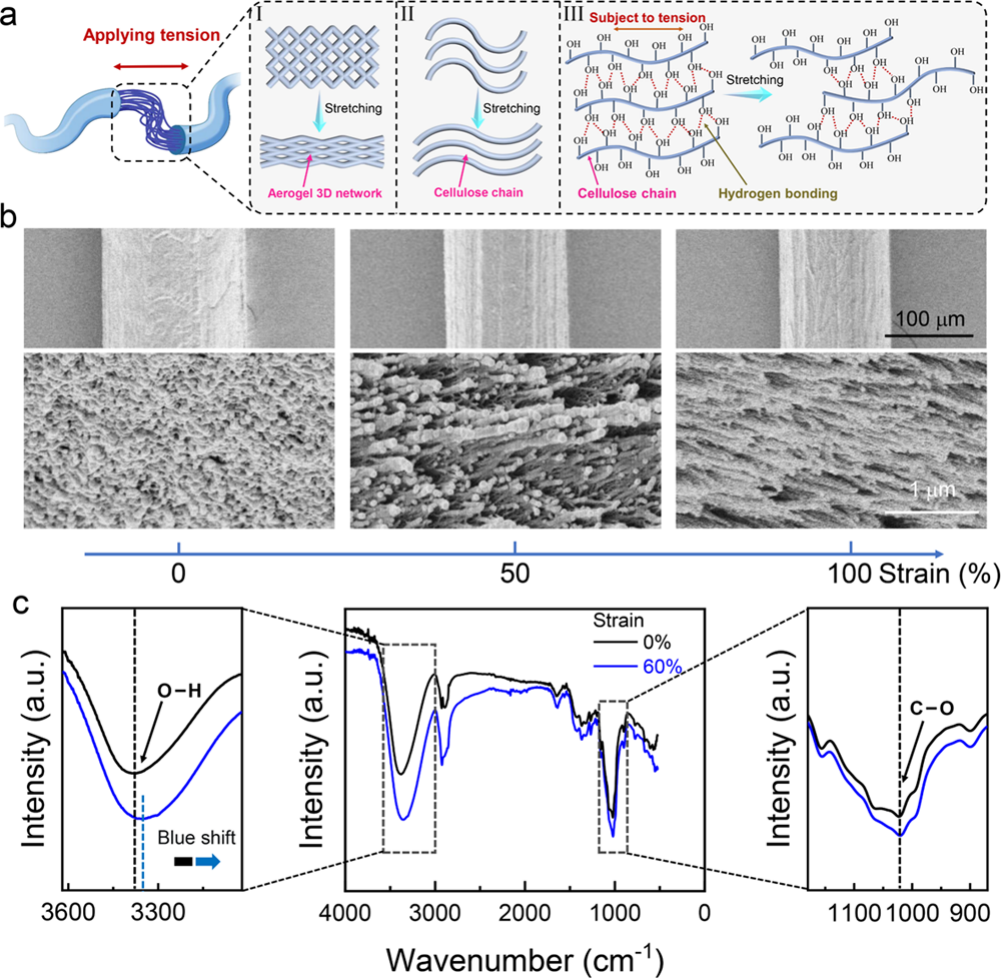
Toughening mechanisms of SAFs. (a) The proposed fracture mechanism of the supertough nanoporous aerogel fibers considering from a macro perspective to a micro perspective. The stretching of the three-dimensional mesh structure (macroscale), the straightening of the bent polymer chains (microscale), and the chain slip phenomenon occurring from hydrogen bonds formed by the hydroxyl groups on the molecular chains (microscale). (b) Surface (upper) and cross section (lower) SEM images of the cellulose nanoporous aerogel fibers prepared with the same preparation parameters at 0, 50, and 100% strain, respectively. (c) Comparisons of Fourier transform infrared spectroscopy (FTIR) spectra of obtained nanoporous aerogel fibers at 0 and 60% strain, respectively.

In order to elaborate on the supertoughness mechanisms of cellulose nanoporous aerogel fibers, we propose the corresponding supertoughness mechanisms from macroscopic and microscopic dimensions, respectively (Figure 3a). Aerogel fibers are typically nanoporous materials with abundant mesopore space and a high specific surface area, which can be simplified as three-dimensional mesh structures. At the macroscopic scale, when the fibers are stretched by an external force, the mesopores deform along the stretching, which induces the fibers to elongate without breakage (Figure 3a, I). Cellulose is a macromolecular polysaccharide composed of glucose units, a polymeric material with plenty of hydroxyl groups on its molecular chain. Generally, the majority of polymer chains constituting cellulose are in a bent state, and when they are subjected to tension, the bent macromolecular chains will gradually straighten, thus also promoting the elongation of fibers (Figure 3a, II). More importantly, there are a large number of hydroxyl groups on the cellulose polymer chains that can form intramolecular and intermolecular hydrogen bonds. Under tension, relative slip occurs between cellulose polymer chains; during this process, the cascade of events of hydrogen bond breakage and reformation happens before the eventual failure contributes to the toughness of cellulose nanoporous aerogel fibers (Figure 3a, III).^48^

Figure 3b shows the surface and cross-sectional SEM images of nanoporous aerogel fibers under different strains. As a result, the diameter of the fiber becomes slimmer upon stretching, and meanwhile, the mesopores inside the fiber also deform along the extension direction. This result is strong evidence for the supertough mechanism of I in Figure 3a at the relatively macroscopic scale. Similar results were obtained by observing nanoporous aerogel fibers under different tensile strains under polarized light (Figure S18). When the nanoporous aerogel fibers are elongated under tension and horizontally placed (or with 45° rotation), it is observed under polarized light that the fibers gradually become thinner, the brightness of the field of view gradually decreases (or increases), the nanofiber orientation factor increases, and the larger mesopores within the fibers gradually become smaller. With further stretching of fibers, the bent polymer chains gradually straighten subjected to tensile forces, and chain slippage occurs, when old hydrogen bonds break and fresh ones form simultaneously due to the abundance of hydroxyl groups on the cellulose chain. The repeated breaking and reforming of hydrogen bonds prominently enhanced resistance force and energy dissipation during intramolecular sliding, resulting in the material’s high tensile strength and toughness (Figure 3a, III). In addition, we also performed infrared characterization of the aerogel fibers before and after stretching (Figure 3c). It can be observed that the absorption peak of −OH (∼3357 cm^–1^) broadens, and the stretching frequency shifts to lower wave numbers (from ∼3387 to ∼3357 cm^–1^). This indicates the increase in the number of hydrogen bonds under stretching, again manifesting the hydrogen bond breaking and reformation events.

The final supertough nanoporous aerogel fiber exhibits not only high strength and toughness but also low density (∼0.043 g/cm^3^ prepared from 1 wt % cellulose) and high porosity up to 97% (eq. S1). Compared with untreated SAFs, the mechanical properties of the SAFs do not change significantly when they are subjected to high-, low-, or high- and low-temperature treatments (Figure S19). More importantly, the SAFs boast good weaveability and can be easily woven into a variety of fabrics, nets, and mesh bags, prompting the tremendous potential for applications in wearable textiles and devices.

**Figure 4:**
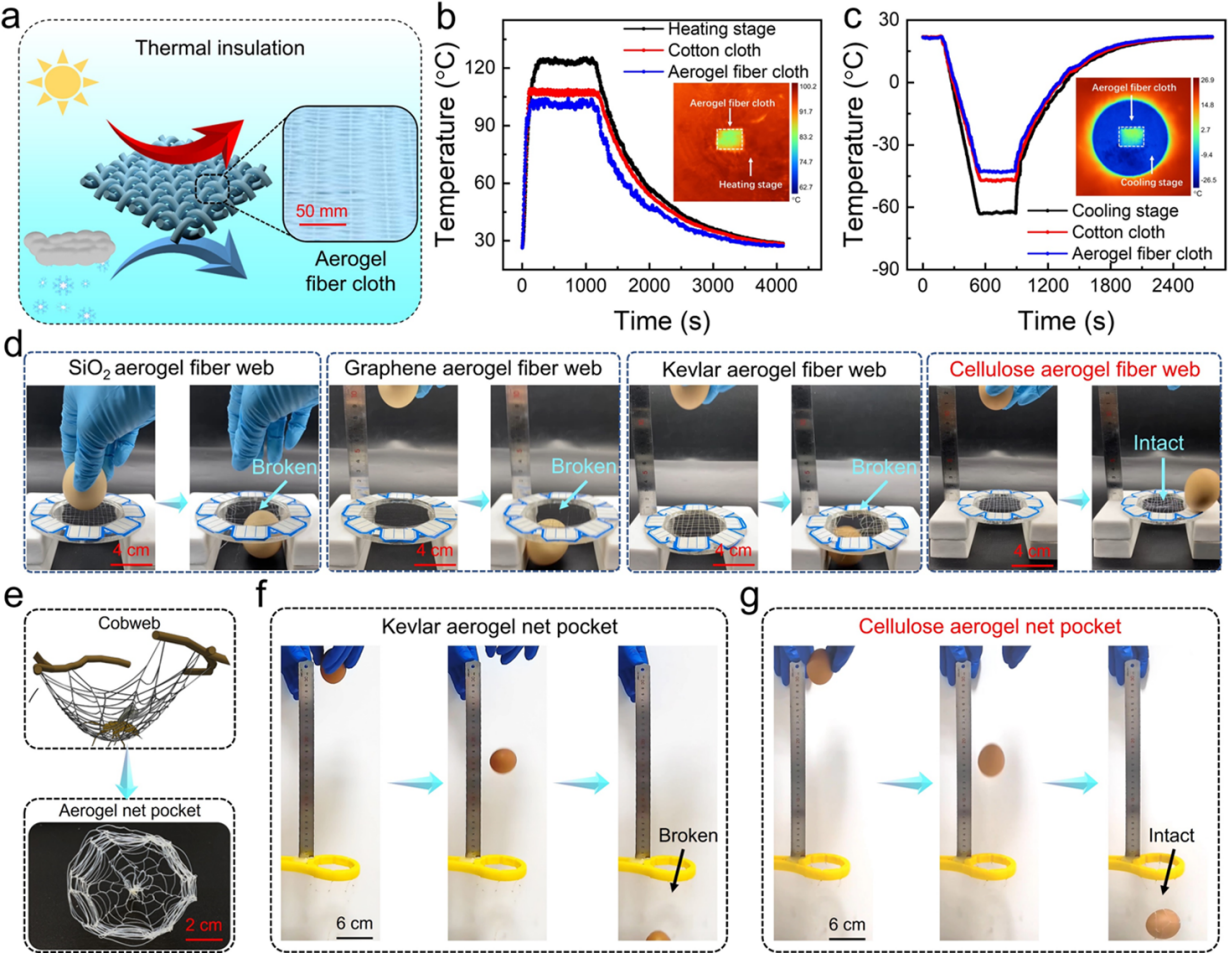
Thermal insulation and toughness performance of SAFs. (a) Schematic thermal insulation of woven cellulose aerogel fiber cloth. (b) Temperature–time curves of the aerogel fiber cloth, cotton cloth, and heating stage surface at high temperatures (∼124 °C). (c) Temperature–time curves of the aerogel fiber cloth, cotton cloth, and cooling stage surface at low temperatures. (d) Photographs of eggs hitting aerogel fiber web composed of different materials. (e) Cobweb-inspired aerogel fiber woven bag. (f,g) Photographs of eggs falling into Kevlar aerogel woven bag (f) and cellulose aerogel woven bag (g), respectively.

First, nanoporous aerogel fibers based on skimmed cotton were woven into cloth to demonstrate the thermal insulation performance at high and low temperatures (Figure 4a). The inset in Figure 4a shows a photograph of a cloth woven from SAFs. It is placed on a hot or cold stage, and its upper and lower surface (hot or cold stage) temperatures are measured and compared with commercially available cotton. Here, thermocouples are used to record the temperature of the aerogel fiber cloth, cotton cloth, hot stage, and cold stage in real time, and an infrared camera is used to record the images when the temperature is stable. Figure 4b,c shows the thermal insulation performance of aerogel fabric and cotton fabric under high- and low-temperature conditions, respectively. The absolute temperature difference (|Δ*T*|) between the upper surface of the cloth and the hot source (or cold source) would represent the thermal insulation property, where a higher |Δ*T*| means better thermal insulation performance at high temperatures (or low temperature).^9^ The |Δ*T*| values for aerogel textile and cotton textile are 24.1 and 13.9 °C under high temperature (or 21 and 15.3 °C under low temperature), respectively, indicating the better thermal insulation property of the aerogel textiles.

To vividly demonstrate the toughness of SAFs, supertough cellulose nanoporous aerogel fibers and conventional nanoporous aerogel fibers (silica, graphene, Kevlar, etc.) were woven into nets, and finally, eggs (mass of 53 ± 2 g) were freely fallen onto variant nets from a certain height (Figure 4d). Results show that silica aerogel net (falling height of ∼2 mm), graphene aerogel net (falling height of ∼87 mm), and Kevlar aerogel net (falling height of ∼120 mm) are easily broken due to the impact of the egg, while supertough cellulose aerogel net remains intact after the egg impact from the maximum height (up to 120 mm). Among the tested nets, the impact experiments tell: cellulose aerogel fibers > Kevlar aerogel fibers > graphene aerogel fibers > silica aerogel fibers. For the most brittle silica aerogel fibers, the woven bag is easily broken even if the egg is located at a height of only 10 mm. This interesting experiment vividly depicts the advantages of supertough nanoporous aerogel fibers over conventional nanoporous aerogel fibers in terms of toughness (Movie S1).

Additionally, to further demonstrate the weaveability of supertough nanoporous aerogel fibers, we imitate the spider web in nature by weaving supertough cellulose aerogel fibers into a bag and taking Kevlar aerogel fibers as a comparison (Figure 4e). This indicates that they both possess a good weaveability performance. Then, an egg (mass of 53 ± 2 g) was dropped from a height of ∼30 cm into as-prepared bags in a free-fall manner (Figure 4f,g). Consequently, the Kevlar aerogel bag was damaged by the impact of an egg, while the supertough aerogel fibers played a good cushioning role to catch the egg and remained intact (Movie S2). This experiment once again demonstrates that the supertough cellulose nanoporous aerogel fibers can resist sudden impact and dissipate energy originating from the intercellulose fiber sliding.

## Conclusions

This work demonstrates a strategy to achieve supertough nanoporous aerogel fibers initially started from ionic-liquid-dissociated cellulose using a wet-spinning methodology. We rationalize the mechanical properties of nanoporous aerogel fibers by tuning the cellulose mass fraction and drying approach. The obtained cellulose nanoporous aerogel fibers possess high tensile strength (up to 30 MPa) without compromising toughness (as high as 21.85 MJ/m^3^). On one hand, the good mechanical strength of cellulose nanoporous aerogel fibers is realized by assembling cellulose macromolecules into nanofibers with *in situ* formed orientation and increasing the cross-linking points among hydroxyl groups attached to the cellulose skeleton. The anisotropy of the nanoporous cellulose aerogel fibers is vividly evident from Herman’s orientation factor by the azimuthal scan of wide-angle X-ray scattering. On the other hand, the compelling toughness of nanoporous cellulose aerogel fibers is attributed to the collective effect of the deformable three-dimensional aerogel mesoporous network, stretchable cellulose polymer chains, and a series of hydrogen bonds’ breakage and reformation on the molecular chains under tension. SEM images and infrared spectra provide strong evidence of the toughening mechanism of cellulose nanoporous aerogel fibers. To our knowledge, the supertough cellulose aerogel fibers can address the conflict between strength and toughness, overweighing other aerogel materials (film/fibers) reported in the literature. Furthermore, the nanoporous aerogel fibers are demonstrated with the desired weavability, thermal insulation performance, and mechanical robustness under transient impact. The strategy in this work can be generally applicable to a myriad of other material systems; combined with the readily available source of cellulose and its biodegradable properties, fertile possibilities could spur the development of wearable textiles, lightweight thermal insulation devices, soft electronics, and flexible devices toward vast applications.

## Methods

### Materials

Skimmed cotton balls are purchased from Qingdao Hainuo Biological Engineering Co. Ionic liquid (1-allyl-3-methylimidazole chloride) was obtained from Anhui Zesheng Technology Co., Ltd. Ethanol was purchased from Sinopharm Chemical Reagent Co. All chemicals were used without further purification. DI water was collected from a Milli-Q water purification system (Millipore).

### Preparation of Cellulose/Ionic Liquid Solution

First, 0.06–0.38 g of skimmed cotton balls were added to 6 g of ionic liquids, which were magnetically stirred for 8–168 h at 80 °C temperature to form a series of solutions of cellulose/ionic liquid with different concentrations (1–6 wt %). The resulting cellulose/ionic liquid solution is dark yellow in color and is not considered completely dissolved until no fine fibers are observed in the sunlight.

### Preparation of Cellulose Hydrogel Fibers and Cellulose Aerogel Fibers

The resulting cellulose/ionic liquid solution was extruded from a pump-controlled syringe into a coagulation bath. The diameter of the fibers is controlled by changing the needle with different diameters, which is connected to the syringe through a section of the transparent catheter. The chosen coagulation bath was anhydrous ethanol. There is a collection device in the coagulation bath to collect the fibers, while the speed of the rotary table under the coagulation bath can be adjusted to achieve the purpose of drawing the fibers. Finally, the cellulose hydrogel fibers are obtained by transferring the previously obtained gel fibers to water and washing them to remove the residual ionic liquid.

The resulting hydrogel fibers were immersed in anhydrous ethanol for solvent replacement. Multiple solvent replacements are performed to ensure the complete removal of impurities from the fiber. Subsequently, supercritical CO_2_ drying was applied to obtain the final cellulose aerogel fibers.

### Characterization

The rheological behaviors of cellulose/ionic liquid solution were carried out on a HAAKE RheoStress 6000 (Thermo Scientific) at 25 °C. Fourier transform infrared (FTIR) spectra were recorded on a Nicolet 6700 FT-IR spectrometer over a wavenumber range of 500–4000 cm^–1^. The morphology and elemental mapping of aerogel fibers were inspected with a field emission scanning electron microscope (Hitachi S-4800) at an acceleration voltage of 10 kV. The specific surface area of the cellulose aerogel fibers was determined at 77 K by the Brunauer–Emmett–Teller (BET) method. X-ray diffraction (XRD) patterns of cellulose aerogel fibers were collected on a D8 Advance Bruker AXS diffractometer with Cu Kα generated at 40 kV and 40 mA. The orientation of the cellulose aerogel fibers is measured by small-angle X-ray scattering (SAXS, Nano STAR, Bruker-AXS). Mechanical properties were tested in tensile mode by an electronic universal testing machine (Instron 3365) with a gauge length of 10 mm at a loading rate of 1 mm/min. A minimum of five samples per condition was tested to obtain statistically reliable values. The infrared images were taken with a MinIR camera (M1100150). The temperature was measured and recorded via a thermal couple and Keysight 34970 Data Acquisition.

## Supporting Information Available

Discussions of porosity measurements and orientation measurements, photographs, NMR hydrogen spectra, FTIR spectra, TEM images, EDS spectrum, photographs in polarized light, SEM images, XRD patterns, TG curves, pore size distribution curves, contact angle images, schematic diagrams and stress–strain curves, and tables of the properties for typical aerogel fibers or films from reported literature (PDF)
Movie S1. Comparison of strength and toughness of aerogel fibers made with different materials (silica, graphene, Kevlar, and cellulose) (MP4)
Movie S2. An experiment of catching a dropped egg from a certain height using a net pocket made of aerogel fibers woven from different materials (Kevlar and cellulose) (MP4)

## References

[ref1] Li X.; Dong G.; Liu Z.; Zhang X. Polyimide Aerogel Fibers with Superior Flame Resistance, Strength, Hydrophobicity, and Flexibility Made via a Universal Sol-Gel Confined Transition Strategy. ACS Nano 2021, 15, 4759–4768. 10.1021/acsnano.0c09391. 33636972

[ref2] Liu Y.; Zhang Y.; Xiong X.; Ge P.; Wu J.; Sun J.; Wang J.; Zhuo Q.; Qin C.; Dai L. Strategies for Preparing Continuous Ultraflexible and Ultrastrong Poly (Vinyl Alcohol) Aerogel Fibers with Excellent Thermal Insulation. Macromol. Mater. Eng. 2021, 306, 2100399 10.1002/mame.202100399.

[ref3] Li M.; Chen X.; Li X.; Dong J.; Zhao X.; Zhang Q. Controllable Strong and Ultralight Aramid Nanofiber-Based Aerogel Fibers for Thermal Insulation Applications. Adv. Fiber Mater. 2022, 4, 1267–1277. 10.1007/s42765-022-00175-2.

[ref4] Xue T.; Zhu C.; Feng X.; Wali Q.; Fan W.; Liu T. Polyimide Aerogel Fibers with Controllable Porous Microstructure for Super-Thermal Insulation Under Extreme Environments. Adv. Fiber Mater. 2022, 4, 1118–1128. 10.1007/s42765-022-00145-8.

[ref5] Pico D.; Meyer E.; Luking A.; Milow B.; Gries T. SILICA-AERO. Production of Lightweight Silica Aerogel Fibers for Excellent Heat Insulating Application. Chem. Eng. Trans. 2017, 60, 91–96. 10.3303/CET1760016.

[ref6] Liu Z.; Lyu J.; Fang D.; Zhang X. Nanofibrous Kevlar Aerogel Threads for Thermal Insulation in Harsh Environments. ACS Nano 2019, 13, 5703–5711. 10.1021/acsnano.9b01094. 31042355

[ref7] Li M.; Gan F.; Dong J.; Fang Y.; Zhao X.; Zhang Q. Facile Preparation of Continuous and Porous Polyimide Aerogel Fibers for Multifunctional Applications. ACS Appl. Mater. Interfaces 2021, 13, 10416–10427. 10.1021/acsami.0c21842. 33595283

[ref8] He H.; Liu J.; Wang Y.; Zhao Y.; Qin Y.; Zhu Z.; Yu Z.; Wang J. An Ultralight Self-Powered Fire Alarm e-Textile Based on Conductive Aerogel Fiber with Repeatable Temperature Monitoring Performance Used in Firefighting Clothing. ACS Nano 2022, 16, 2953–2967. 10.1021/acsnano.1c10144. 35084187

[ref9] Yang H.; Wang Z.; Liu Z.; Cheng H.; Li C. Continuous, Strong, Porous Silk Firoin-Based Aerogel Fibers toward Textile Thermal Insulation. Polymers 2019, 11, 1899 10.3390/polym11111899. 31752126 PMC6918396

[ref10] Zuo X.; Fan T.; Qu L.; Zhang X.; Miao J. Smart Multi-Responsive Aramid Aerogel Fiber Enabled Self-Powered Fabrics. Nano Energy 2022, 101, 107559 10.1016/j.nanoen.2022.107559.

[ref11] Hou Y.; Sheng Z.; Fu C.; Kong J.; Zhang X. Hygroscopic Holey Graphene Aerogel Fibers Enable Highly Efficient Moisture Capture, Heat Allocation and Microwave Absorption. Nat. Commun. 2022, 13, 1227 10.1038/s41467-022-28906-4. 35264594 PMC8907192

[ref12] Wu X.; Hong G.; Zhang X. Electroless Plating of Graphene Aerogel Fibers for Electrothermal and Electromagnetic Applications. Langmuir 2019, 35, 3814–3821. 10.1021/acs.langmuir.8b04007. 30768281

[ref13] Aliev A. E.; Oh J.; Kozlov M. E.; Kuznetsov A. A.; Fang S.; Fonseca A. F.; Ovalle R.; Lima M. D.; Haque M. H.; Gartstein Y. N.; Zhang M.; Zakhidov A. A.; Baughman R. H. Giant-Stroke, Superelastic Carbon Nanotube Aerogel Muscles. Science 2009, 323, 1575–1578. 10.1126/science.1168312. 19299612

[ref14] Maziz A.; Concas A.; Khaldi A.; Stalhand J.; Persson N.-K.; Jager E. W. Knitting and Weaving Artificial Muscles. Sci. Adv. 2017, 3, e1600327 10.1126/sciadv.1600327. 28138542 PMC5266480

[ref15] Bao Y.; Lyu J.; Liu Z.; Ding Y.; Zhang X. Stiffness-Directed Fabricating of Kevlar Aerogel-Confined Organic Phase-Change Fibers. ACS Nano 2021, 15, 15180–15190. 10.1021/acsnano.1c05693. 34423639

[ref16] Li Y.; Zhang X. Electrically Conductive, Optically Responsive, and Highly Orientated Ti_3_C_2_Tx MXene Aerogel Fibers. Adv. Funct. Mater. 2022, 32, 2107767 10.1002/adfm.202107767.

[ref17] Xu Z.; Zhang Y.; Li P.; Gao C. Strong, Conductive, Lightweight, Neat Graphene Aerogel Fibers with Aligned Pores. ACS Nano 2012, 6, 7103–7113. 10.1021/nn3021772. 22799441

[ref18] Du Y.; Zhang X.; Wang J.; Liu Z.; Zhang K.; Ji X.; You Y.; Zhang X. Reaction-Spun Transparent Silica Aerogel Fibers. ACS Nano 2020, 14, 11919–11928. 10.1021/acsnano.0c05016. 32902257

[ref19] Meng S.; Zhang J.; Xu W.; Chen W.; Zhu L.; Zhou Z.; Zhu M. Structural Control of Silica Aerogel Fibers for Methylene Blue Removal. Science ci. China: Technol. Sci. 2019, 62, 958–964. 10.1007/s11431-018-9389-7.

[ref20] Li G.; Hong G.; Dong D.; Song W.; Zhang X. Multiresponsive Graphene-Aerogel-Directed Phase-Change Smart Fibers. Adv. Mater. 2018, 30, 1801754 10.1002/adma.201801754. 29904953

[ref21] Chen Y.; Zhou L.; Chen L.; Duan G.; Mei C.; Huang C.; Han J.; Jiang S. Anisotropic Nanocellulose Aerogels with Ordered Structures Fabricated by Directional Freeze-Drying for Fast Liquid Transport. Cellulose 2019, 26, 6653–6667. 10.1007/s10570-019-02557-z.

[ref22] Chen Y.; Zhang L.; Mei C.; Li Y.; Duan G.; Agarwal S.; Greiner A.; Ma C.; Jiang S. Wood-Inspired Anisotropic Cellulose Nanofibril Composite Sponges for Multifunctional Applications. ACS Appl. Mater. Interfaces 2020, 12, 35513–35522. 10.1021/acsami.0c10645. 32672439

[ref23] Fu C.; Sheng Z.; Zhang X. Laminated Structural Engineering Strategy toward Carbon Nanotube-Based Aerogel Films. ACS Nano 2022, 16, 9378–9388. 10.1021/acsnano.2c02193. 35587451 PMC9245345

[ref24] Liu Z.; Lyu J.; Ding Y.; Bao Y.; Sheng Z.; Shi N.; Zhang X. Nanoscale Kevlar Liquid Crystal Aerogel Fibers. ACS Nano 2022, 16, 15237–15248. 10.1021/acsnano.2c06591. 36053080 PMC9527790

[ref25] Chen Y.; Li S.; Li X.; Mei C.; Zheng J.; E S.; Duan G.; Liu K.; Jiang S. Liquid Transport and Real-Time Dye Purification via Lotus Petiole-Inspired Long-Range-Ordered Anisotropic Cellulose Nanofibril Aerogels. ACS Nano 2021, 15, 20666–20677. 10.1021/acsnano.1c10093. 34881863

[ref26] Chen Y.; Luo H.; Guo H.; Liu K.; Mei C.; Li Y.; Duan G.; He S.; Han J.; Zheng J.; E S.; Jiang S. Anisotropic Cellulose Nanofibril Composite Sponges for Electromagnetic Interference Shielding with Low Reflection Loss. Carbohyd. Polym. 2022, 276, 118799 10.1016/j.carbpol.2021.118799. 34823805

[ref27] Zou Y.; Zhao J.; Zhu J.; Guo X.; Chen P.; Duan G.; Liu X.; Li Y. A Mussel-Inspired Polydopamine-Filled Cellulose Aerogel for Solar-Enabled Water Remediation. ACS Appl. Mater. Interfaces 2021, 13, 7617–7624. 10.1021/acsami.0c22584. 33538165

[ref28] Zhang H.; Wu J.; Zhang J.; He J. 1-Allyl-3-methylimidazolium Chloride Room Temperature Ionic Liquid: A New and Powerful Nonderivatizing Solvent for Cellulose. Macromolecules 2005, 38, 8272–8277. 10.1021/ma0505676.

[ref29] Kuang Q.-L.; Zhao J.-C.; Niu Y.-H.; Zhang J.; Wang Z.-G. Celluloses in An Ionic Liquid: the Rheological Properties of the Solutions Spanning the Dilute and Semidilute Regimes. J. Phys. Chem. B 2008, 112, 10234–10240. 10.1021/jp804167n. 18661932

[ref30] Song H.; Zhang J.; Niu Y.; Wang Z. Phase Transition and Rheological Behaviors of Concentrated Cellulose/Ionic Liquid Solutions. J. Phys. Chem. B 2010, 114, 6006–6013. 10.1021/jp1013863. 20405880

[ref31] Sheppard S. The Dispersion of Cellulose and Cellulose Derivatives. J. Phys. Chem. 1930, 34, 1041–1052. 10.1021/j150311a012.

[ref32] Habibi Y.; Lucia L. A.; Rojas O. J. Cellulose Nanocrystals: Chemistry, Self-Assembly, and Applications. Chem. Rev. 2010, 110, 3479–3500. 10.1021/cr900339w. 20201500

[ref33] He W.; Zheng J.; Dong W.; Jiang S.; Lou G.; Zhang L.; Du W.; Li Z.; Li X.; Chen Y. Efficient Electromagnetic Wave Absorption and Joule Heating via Ultra-Light Carbon Composite Aerogels Derived from Bimetal-Organic Frameworks. Chem. Eng. J. 2023, 459, 141677 10.1016/j.cej.2023.141677.

[ref34] Yao K.; Song C.; Fang H.; Wang F.; Chen L.; Jiang S.; Zha G.; Hou H. Freezing-Extraction/Vacuum-Drying Method for Robust and Fatigue-Resistant Polyimide Fibrous Aerogels and Their Composites with Enhanced Fire Retardancy. Engineering 2023, 21, 152–161. 10.1016/j.eng.2021.08.024.

[ref35] Jiang S.; Cheong J. Y.; Nam J. S.; Kim I.-D.; Agarwal S.; Greiner A. High-Density Fibrous Polyimide Sponges with Superior Mechanical and Thermal Properties. ACS Appl. Mater. Interfaces 2020, 12, 19006–19014. 10.1021/acsami.0c02004. 32216283

[ref36] Qian Y.-q.; Han N.; Bo Y.-w.; Tan L.-l.; Zhang L.-f.; Zhang X.-x. Homogeneous Synthesis of Cellulose Acrylate-g-Poly (*n*-alkyl acrylate) Solid-Solid Phase Change Materials via Free Radical Polymerization. Carbohyd. Polym. 2018, 193, 129–136. 10.1016/j.carbpol.2018.03.057. 29773364

[ref37] Zhang J.; Feng L.; Wang D.; Zhang R.; Liu G.; Cheng G. Thermogravimetric Analysis of Lignocellulosic Biomass with Ionic Liquid Pretreatment. Bioresource Technol. 2014, 153, 379–382. 10.1016/j.biortech.2013.12.004. 24365118

[ref38] Huang J.; Li J.; Xu X.; Hua L.; Lu Z. *In Situ* Loading of Polypyrrole onto Aramid Nanofiber and Carbon Nanotube Aerogel Fibers as Physiology and Motion Sensors. ACS Nano 2022, 16, 8161–8171. 10.1021/acsnano.2c01540. 35481375

[ref39] Wang Y.; Cui Y.; Shao Z.; Gao W.; Fan W.; Liu T.; Bai H. Multifunctional Polyimide Aerogel Textile Inspired by Polar Bear Hair for Thermoregulation in Extreme Environments. Chem. Eng. J. 2020, 390, 124623 10.1016/j.cej.2020.124623.

[ref40] Wang Z.; Yang H.; Li Y.; Zheng X. Robust Silk Fibroin/Graphene Oxide Aerogel Fiber for Radiative Heating Textiles. ACS Appl. Mater. Interfaces 2020, 12, 15726–15736. 10.1021/acsami.0c01330. 32167746

[ref41] Li J.; Wang J.; Wang W.; Zhang X. Symbiotic Aerogel Fibers Made via In-Situ Gelation of Aramid Nanofibers with Polyamidoxime for Uranium Extraction. Molecules 2019, 24, 1821 10.3390/molecules24091821. 31083542 PMC6539675

[ref42] Yang S.; Xie C.; Qiu T.; Tuo X. The Aramid-Coating-on-Aramid Strategy toward Strong, Tough, and Foldable Polymer Aerogel Films. ACS Nano 2022, 16, 14334–14343. 10.1021/acsnano.2c04572. 35994616

[ref43] Li T.; Song J.; Zhao X.; Yang Z.; Pastel G.; Xu S.; Jia C.; Dai J.; Chen C.; Gong A.; Jiang F.; Yao Y.; Fan T.; Yang B.; Wågberg L.; Yang R.; Hu L. Anisotropic, Lightweight, Strong, and Super Thermally Insulating Nanowood with Naturally Aligned Nanocellulose. Sci. Adv. 2018, 4, eaar3724 10.1126/sciadv.aar3724. 29536048 PMC5844708

[ref44] Chen Y.; Zhang C.; Tao S.; Chai H.; Xu D.; Li X.; Qi H. High-Performance Smart Cellulose Nanohybrid Aerogel Fibers as a Platform toward Multifunctional Textiles. Chem. Eng. J. 2023, 466, 143153 10.1016/j.cej.2023.143153.

[ref45] Cai J.; Liu S.; Feng J.; Kimura S.; Wada M.; Kuga S.; Zhang L. Cellulose-Silica Nanocomposite Aerogels by *in Situ* Formation of Silica in Cellulose Gel. Angew. Chem. 2012, 124, 2118–2121. 10.1002/ange.201105730. 22275132

[ref46] Lee M.; Hyman W. Modeling of Failure Mode in Knee Ligaments Depending on the Strain Rate. BMC musculoskel. dis. 2002, 3, 3 10.1186/1471-2474-3-3. PMC65677 11860613

[ref47] Vázquez-Rodríguez J.; Flores-Johnson E.; Herrera-Franco P.; Gonzalez-Chi P. Photoelastic and Numerical Analyses of the Stress Distribution around a Fiber in a Pull-Out Test for a Thermoplastic Fiber/Epoxy Resin Composite. Polym. Composite. 2018, 39, E2397–E2406. 10.1002/pc.24709.

[ref48] Fan L.; Zeng Z.; Zhu R.; Liu A.; Che H.; Huo M. Polymerization-Induced Self-Assembly Toward Micelle-Crosslinked Tough and Ultrastretchable Hydrogels. Chem. Mater. 2022, 34, 6408–6419. 10.1021/acs.chemmater.2c01001.

