# Supplementary material for: Ionic Liquid Directed Spinning of Cellulose Aerogel Fibers with Superb Toughness for Weaved Thermal Insulation and Transient Impact Protection
Source: ACS Nano. 2023 Sep 12;17(18):18411–20. doi: 10.1021/acsnano.3c05894 (PMC10540260; doi:10.1021/acsnano.3c05894)
Supplement: Supplementary file 1 — nn3c05894_si_001.pdf [file nn3c05894_si_001.pdf]

Supporting Information

Ionic Liquid Directed Spinning of Cellulose Aerogel Fibers with Superb Toughness for Weaved Thermal Insulation and Transient Impact Protection

Zhongsheng Liu,[a†] *Zhizhi Sheng,*[a†] *Yaqian Bao,*[a] *Qingqing Cheng,*[a] *Pei-xi Wang,*[a] *Zengwei Liu,*[a] *and Xuetong Zhang*[a,b]*

[a]Suzhou Institute of Nano-tech and Nano-bionics, Chinese Academy of Sciences, Suzhou 215123, P. R. China. E-mail: xtzhang2013@sinano.ac.cn

[b]Department of Surgical Biotechnology, Division of Surgery & Interventional Science, University College London, London, NW3 2PF, UK. E-mail: xuetong.zhang@ucl.ac.uk

[†]These authors contributed equally to this work

1. Porosity for cellulose aerogel fibers

Porosity refers to the extent of empty spaces or voids present in a material, and is expressed as a ratio of the volume of these voids to the total volume of the material, between 0% and 100%:

$$\Phi = \frac{\rho_0 - \rho}{\rho_0} \times 100\% \qquad \text{(S1)}$$

where Φ is porosity, ρ_0 is the material density, and ρ is the density of aerogel fibers.

2. Orientation measurements for cellulose aerogel fibers

Herman's orientation factor (*f*) was calculated to describe the degree of orientation of the cellulose nanofibers relative to the fiber axis direction using Equation (S2):[1]

$$f = \frac{3\langle \cos^2 \emptyset \rangle - 1}{2} \qquad \text{(S2)}$$

where the mean-square cosine is calculated from the scattered intensity *I*(*ø*) by integrating over the azimuthal angle *ø* according to Equation (S3):

$$\langle \cos^2 \emptyset \rangle = \frac{\int_0^{\frac{\pi}{2}} I(\emptyset) \sin\emptyset \cos^2\emptyset d\emptyset}{\int_0^{\frac{\pi}{2}} I(\emptyset) \sin\emptyset d\emptyset} \qquad \text{(S3)}$$

where *ø* is the angle between the fiber axis direction and the cellulose nanofibers. The Herman's orientation factor (*f*) ranges from 0 to 1, where a value of 1 indicates that all nanofibers are perfectly aligned with the fiber axis direction, and a value of 0 indicates a random orientation of the cellulose nanofibers.

3. Supplementary figures

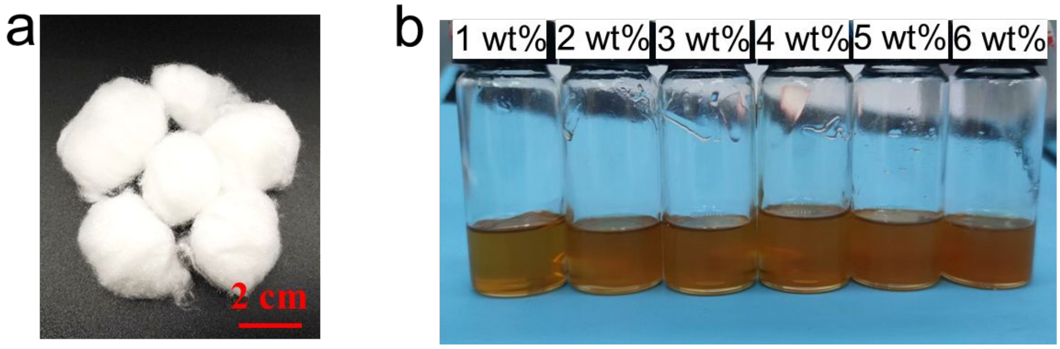

Figure S1. a) Optical picture of defatted cotton as a source of cellulose. b) Optical image of different mass fractions of skimmed cotton dissolved in ionic liquid.

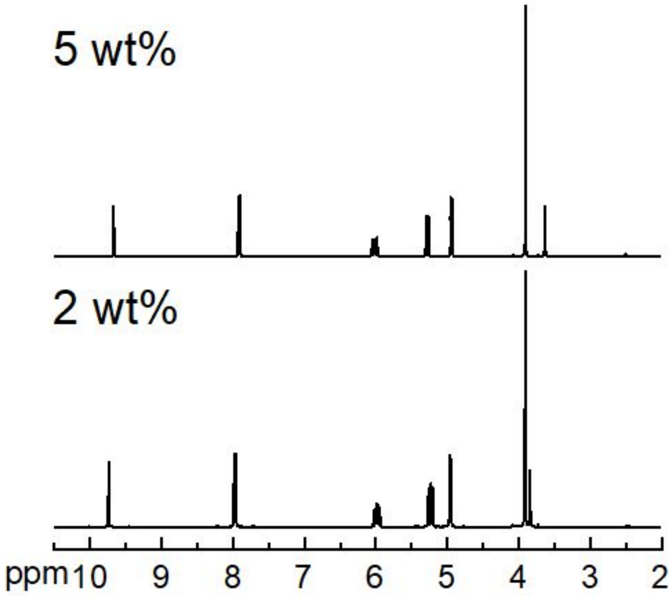

Figure S2. NMR hydrogen spectra of cellulose/ionic liquid solutions with mass fractions of 2 wt% and 5 wt%, respectively.

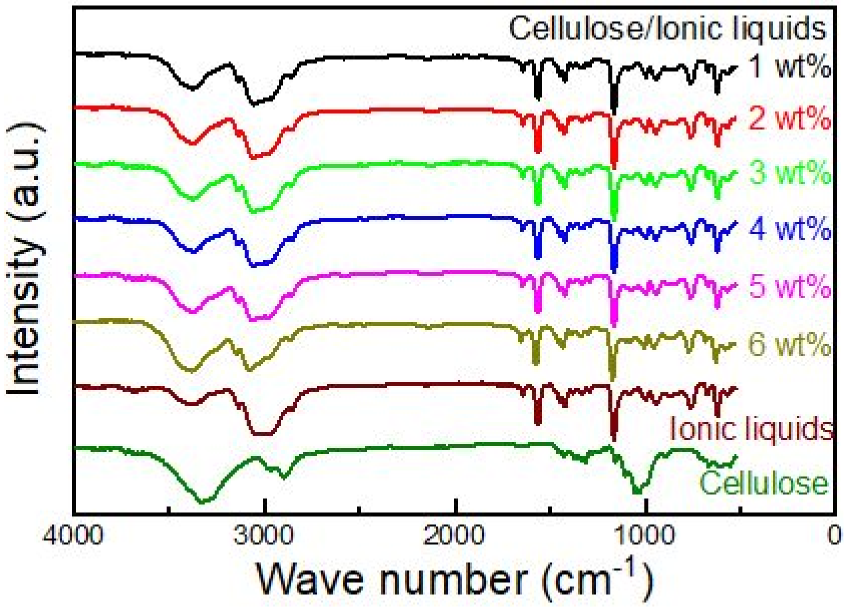

Figure S3. Comparisons of Fourier-transform infrared spectroscopy (FTIR) spectra of obtained cellulose/ionic liquid solution, cellulose, and ionic liquid.

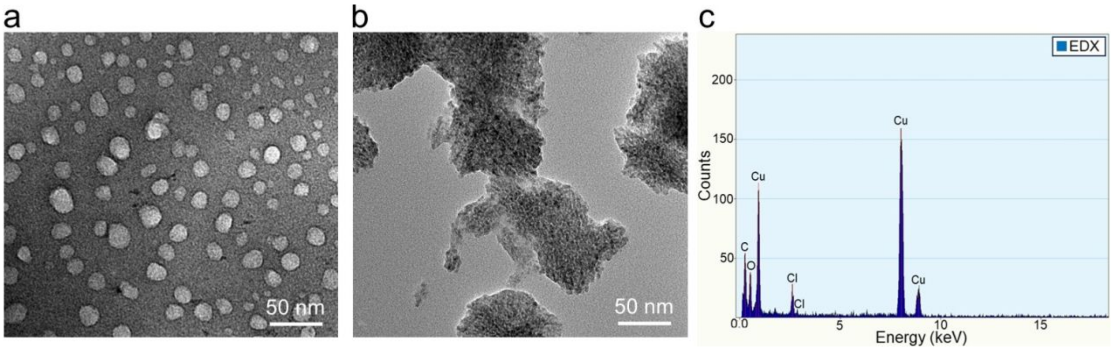

Figure S4. a, b) Transmission electron microscopy (TEM) images of (a) ionic liquids (1-Allyl-3-methylimidazole chloride) and (b) cellulose/ionic liquid solution. c) Energy Dispersive Spectroscopy (EDS) spectra of Figure S4b.

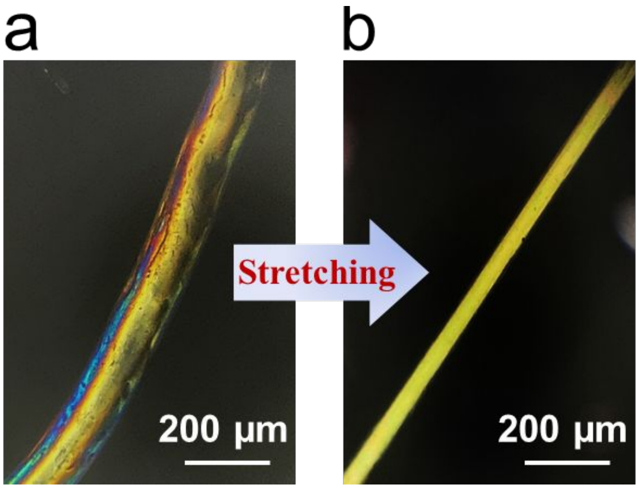

Figure S5. Images of cellulose hydrogel fiber under polarizing microscope before (a) and after stretching (b).

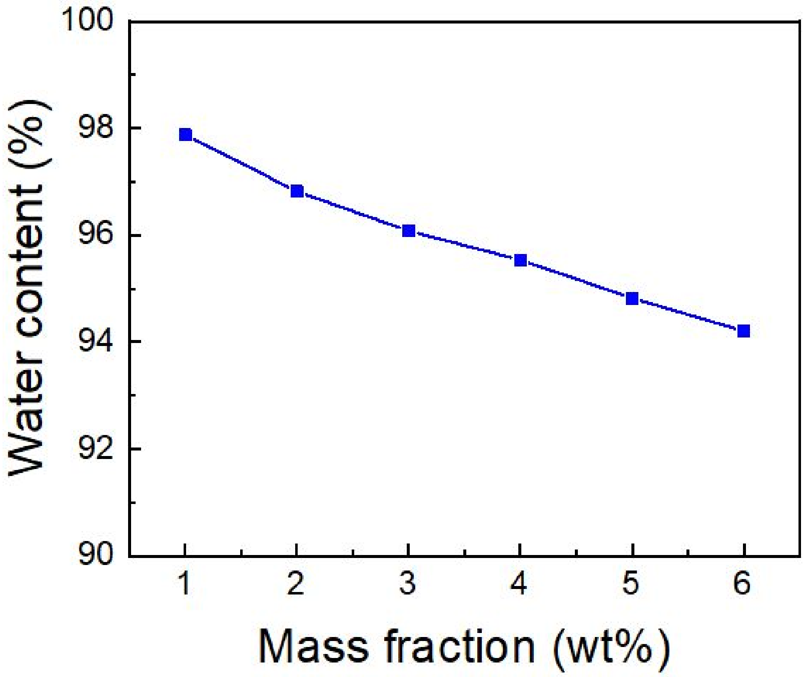

Figure S6. Water content of cellulose hydrogel fibers with different mass fractions.

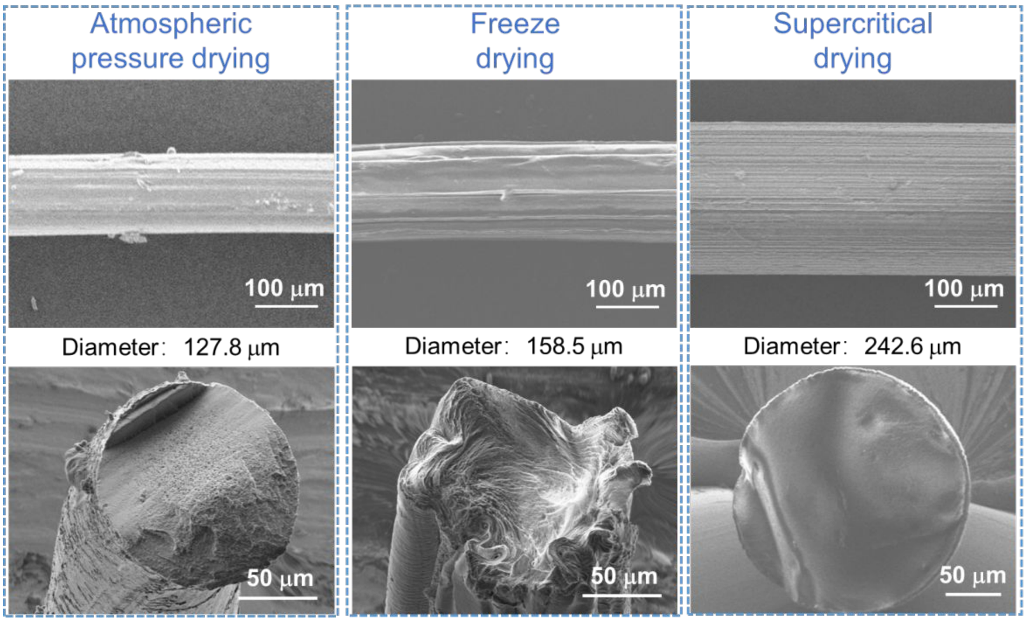

Figure S7. Surface and cross-sectional SEM images of the cellulose aerogel fibers fabricated with different drying methods.

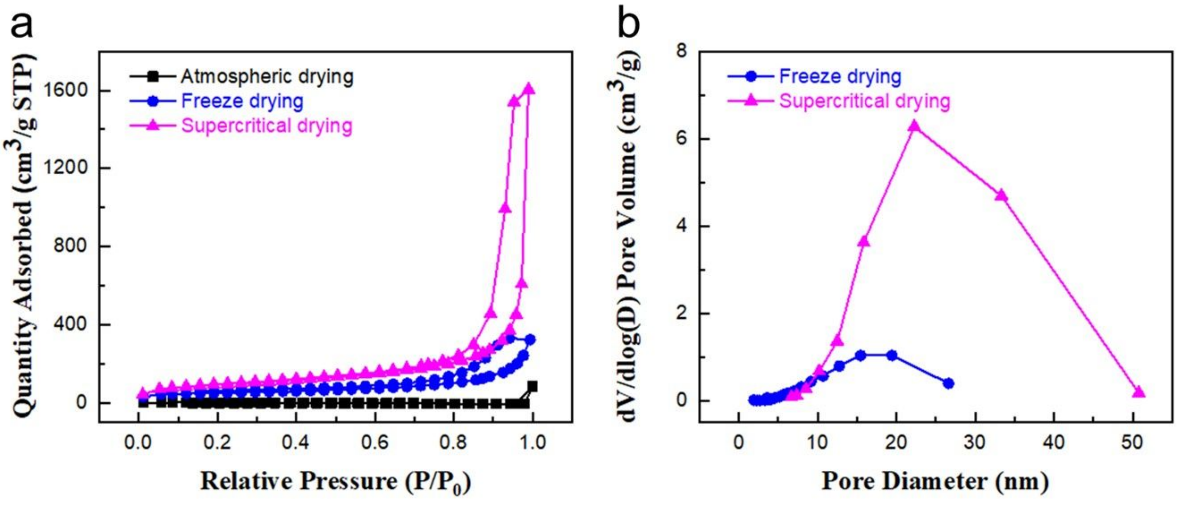

Figure S8. a) Nitrogen adsorption-desorption isotherms of 5 wt% cellulose aerogel fibers obtained with different drying methods. b) Pore volume of the 5 wt% cellulose aerogel fibers obtained by freeze drying and supercritical drying.

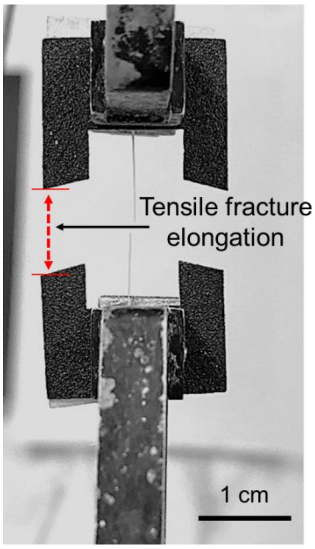

Figure S9. A photo of the tensile fracture of a cellulose aerogel fiber after testing stress-strain curve.

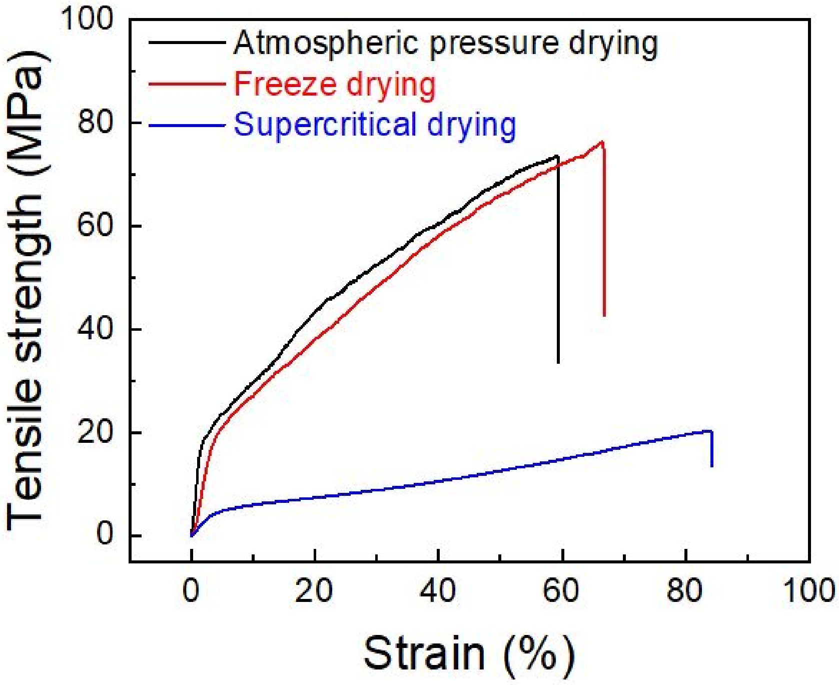

Figure S10. Stress-strain curves of the cellulose aerogel fibers dried by different methods.

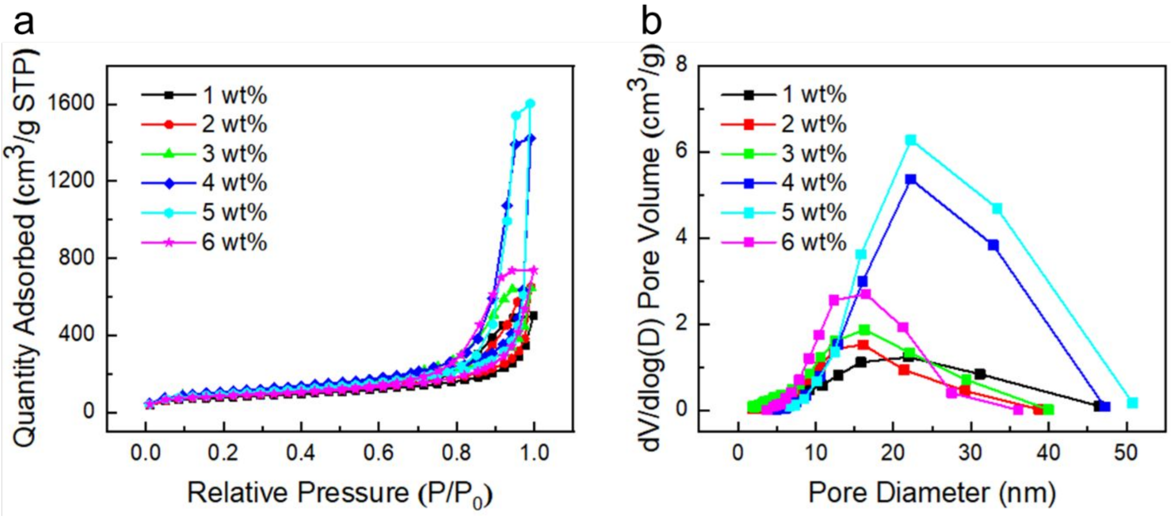

Figure S11. a) Nitrogen adsorption-desorption isotherms of 1-6 wt% cellulose aerogel fibers. b) Pore volume of the 1-6 wt% cellulose aerogel fibers.

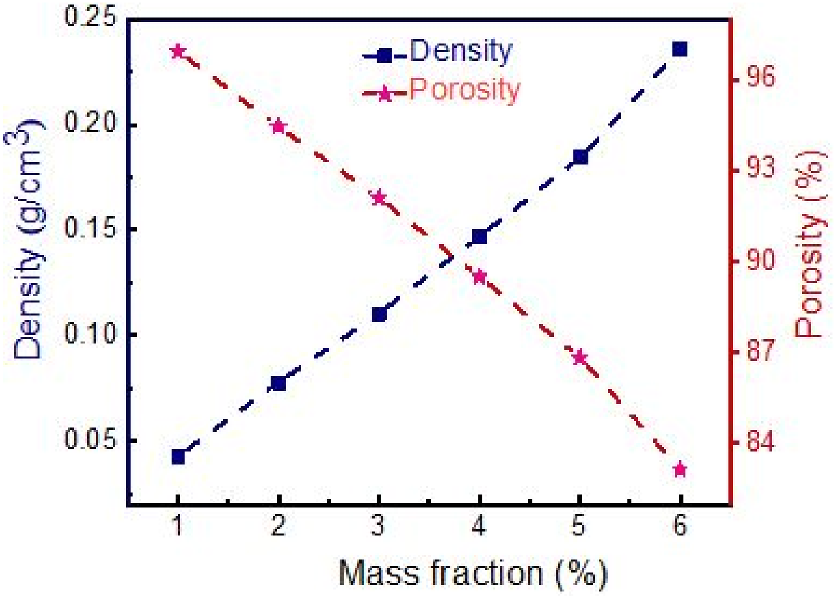

Figure S12. Density and porosity of cellulose aerogel fibers with different mass fractions.

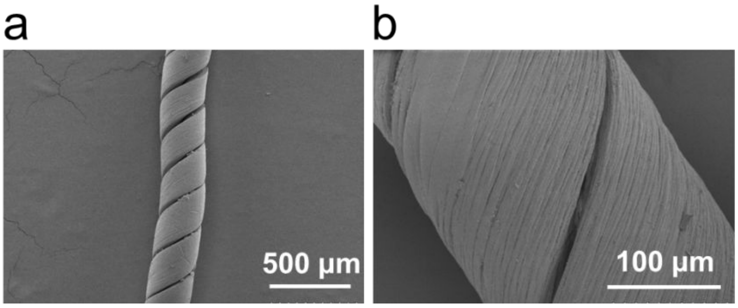

Figure S13. a, b) SEM images of single cellulose aerogel fiber twisted at low (a) and high (b) magnification.

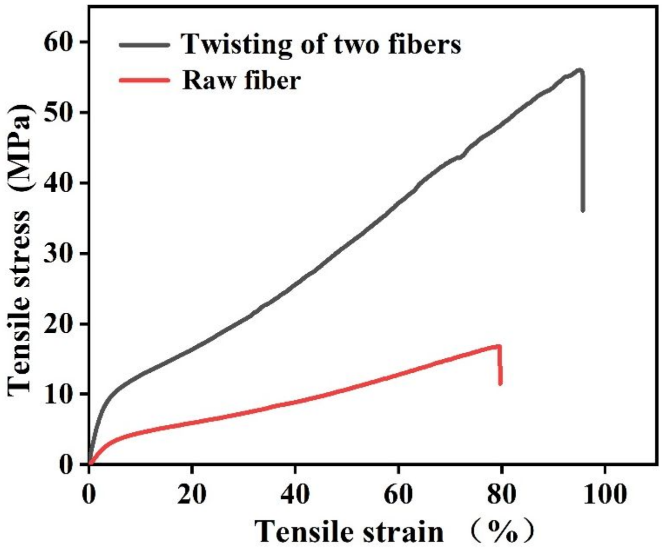

Figure S14. Stress-strain curves of two aerogel fibers after twisting and a single aerogel fiber without twisting.

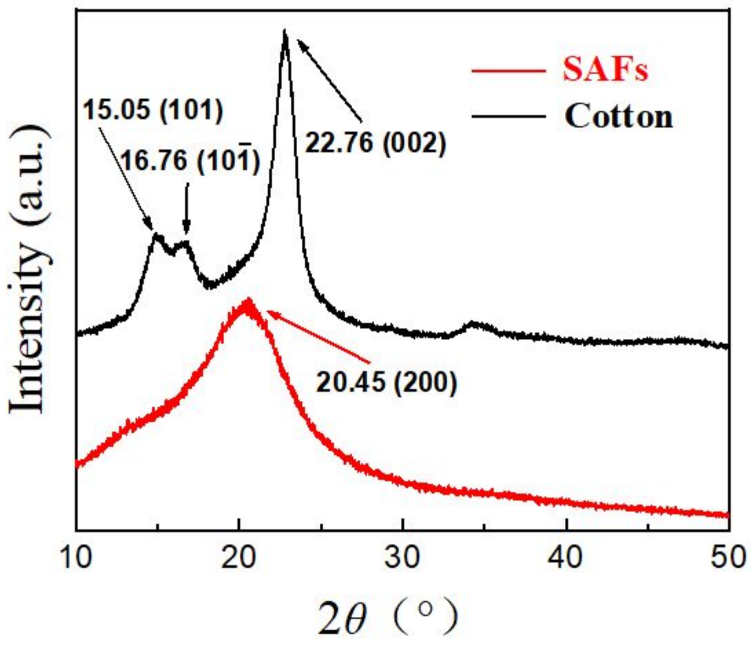

Figure S15. XRD patterns of SAFs and cotton.

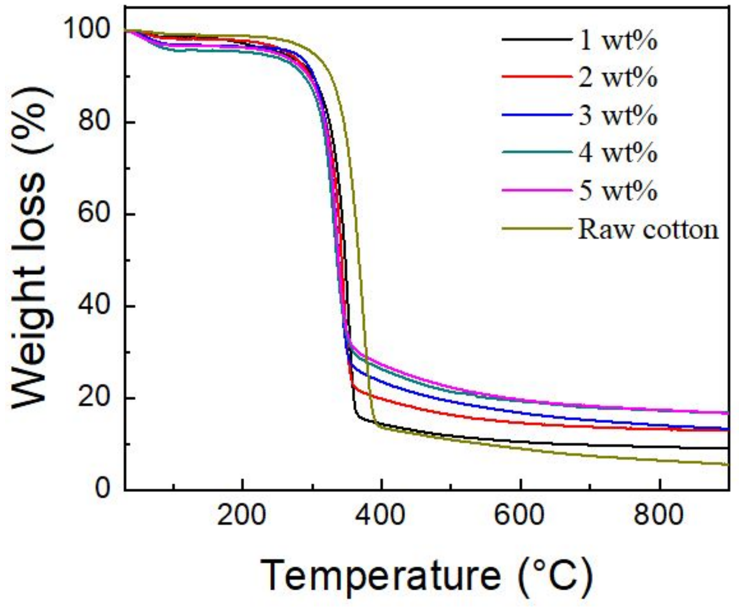

Figure S16. Thermogravimetric (TG) test curves of raw cotton and cellulose aerogel fibers with different mass fractions.

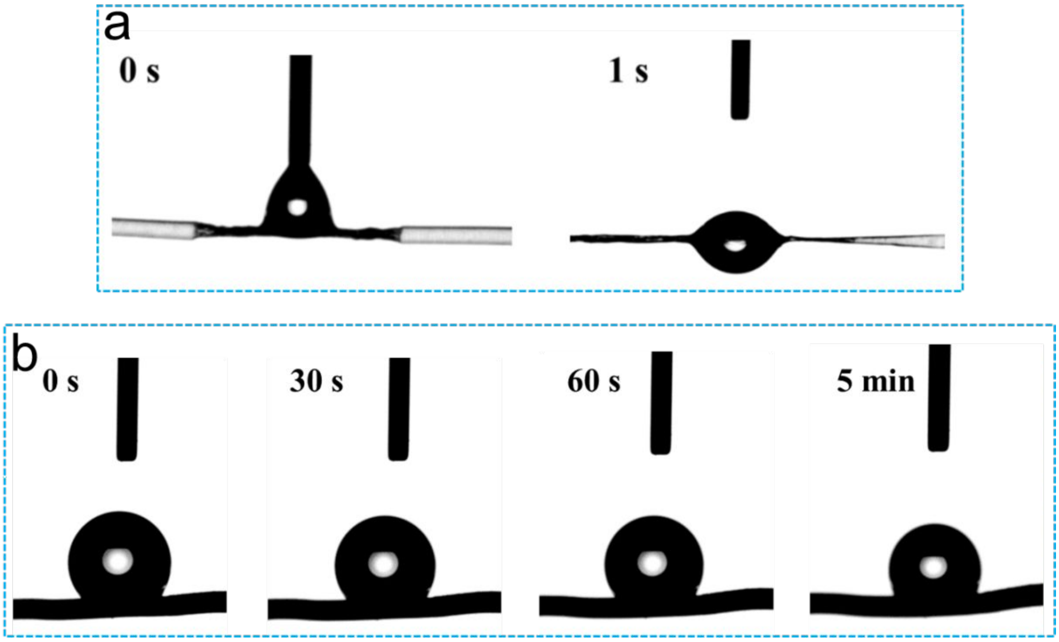

Figure S17. a) Photographs of cellulose aerogel fibers in contact with water. b) photographs of aerogel fibers after hydrophobization by cold plasma at different times in contact with water.

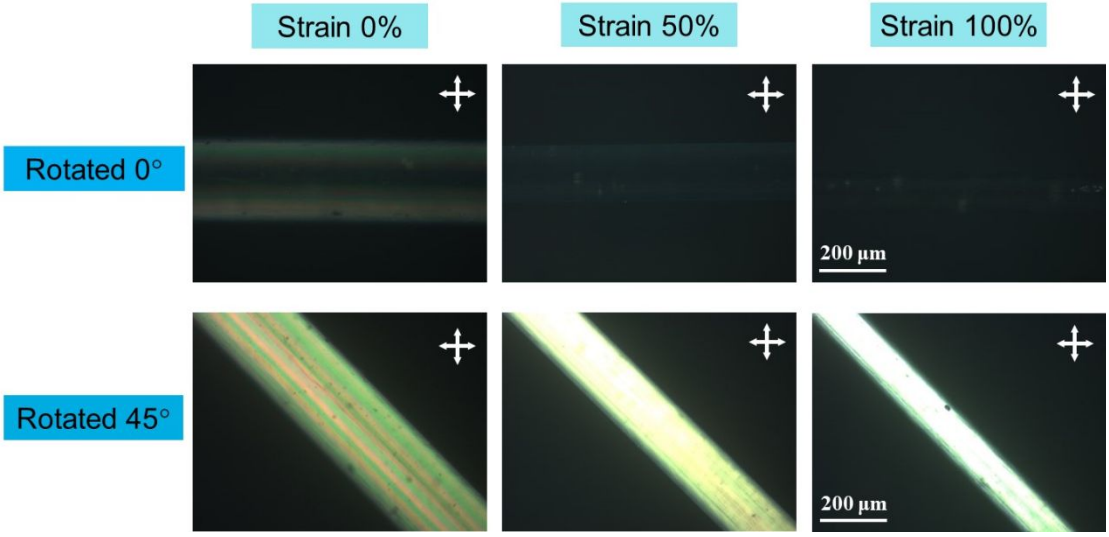

Figure S18. Pictures of cellulose nanoporous aerogel fibers under polarized light at different tensile strains when placed horizontally (rotated 0°) and with 45°rotation.

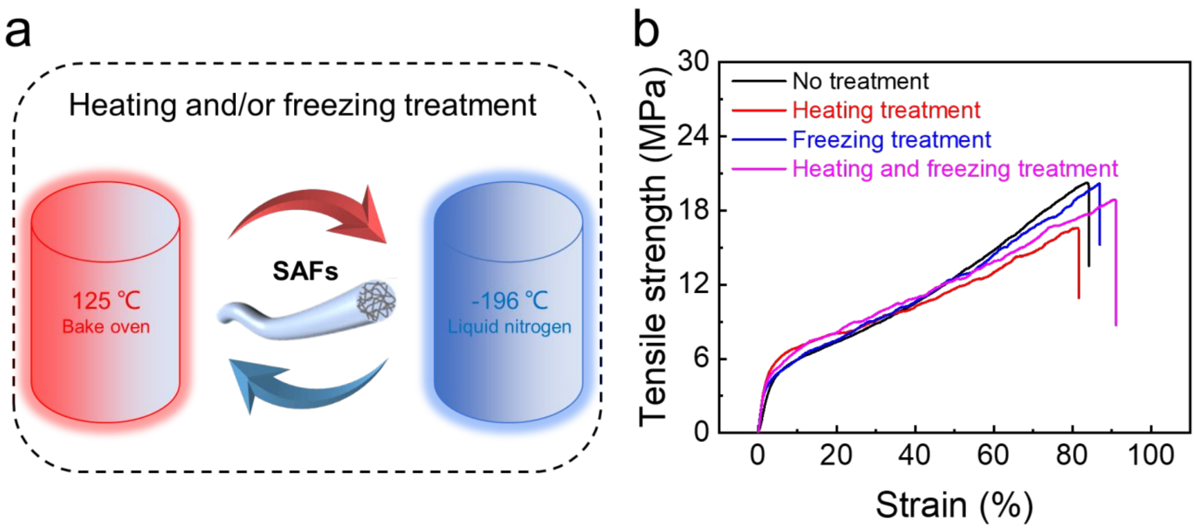

Figure S19. a) Schematic diagram of heat and cold treatment of SAFs. b) Stress-strain curves of SAFs at no treatment, heating treatment, freezing treatment, and heating and freezing treatment.

4. Supplementary Table

Table S1. Summary of the properties for typical aerogel fibers or films from reported literatures.

**Material**	**Journal**	**Year**	**Maximum Strength (MPa)**	**Maximum Elongation (%)**	**Maximum Toughness (MJ/m^3)**	**Literature**
Holey graphene/LiCl	Nature Communications	2022	1.04	10	0.045	1[2]
Nanofibrous Kevlar	ACS Nano	2019	3.25	36	0.63	2[3]
Aramid nanofibers/ Carbon nanotube/ Polypyrrole	ACS Nano	2022	2.88	21.02	0.32	3[4]
Porous Graphene/Paraffin	Advanced Materials	2018	7.54	2.6	0.1	4[5]
$Ti_3C_2T_x$ MXene	Advanced Functional Materials	2022	1.07	0.53	0.004	5[6]
Graphene	ACS Nano	2012	11.17	6.15	0.4	6[7]
Polyimide	ACS Nano	2021	10.67	0.25	0.02	7[8]
Silica	ACS Nano	2020	0.24	3.05	0.003	8[9]
Polyimide	Advanced Fiber Materials	2022	5.25	29.5	0.48	9[10]
Cellulose acetate/Polyacrylic acid	Polymers	2019	2.67	9.14	0.17	10[11]
Poly(Vinyl Alcohol)	Macromolecular Materials and Engineering	2021	8.2	35	2.25	11[12]
Cellulose/Silica nanocomposite	Angewandte Chemie	2012	11.75	57.6	3.91	12[13]

Calcium alginate/Fe_3O_4 nanoparticles/Silver nanowires	ACS Nano	2022	13	31.68	1.8	13[14]
Polyimide	Chemical Engineering Journal	2020	10.64	29.5	2.11	14[15]
Fibroin/Graphene Oxide	ACS Applied Materials & Interfaces	2020	3	8.6	0.18	15[16]
Nanoscale Kevlar	ACS Nano	2022	4.39	44	1.22	16[17]
Polyamidoxime /Aramid nanofiber	Molecules	2019	4.55	24.4	0.61	17[18]
Holocellulose nanofibrils/Cellulos e	Chemical Engineering Journal	2023	23.26	20.7	4.6	18[19]
Cellulose	/	2023	30	107	21.85	This work

5. Supplementary Movies

Movie S1. Comparison of strength and toughness of aerogel fibers made with different materials (silica, graphene, Kevlar, and cellulose).

Movie S2. An experiment of catching a dropped egg from a certain height using a net pocket made of aerogel fibers woven from different materials (Kevlar and cellulose).

6. References

(1) Li, S.; Fan, Z.; Wu, G.; Shao, Y.; Xia, Z.; Wei, C.; Shen, F.; Tong, X.; Yu, J.; Chen, K. Assembly of Nanofluidic MXene Fibers with Enhanced Ionic Transport and Capacitive Charge Storage by Flake Orientation. *ACS Nano* **2021**, *15*, 7821–7832.

(2) Hou, Y.; Sheng, Z.; Fu, C.; Kong, J.; Zhang, X. Hygroscopic Holey Graphene Aerogel Fibers Enable Highly Efficient Moisture Capture, Heat Allocation and Microwave Absorption. *Nat. Commun.* **2022**, *13*, 1227.

(3) Liu, Z.; Lyu, J.; Fang, D.; Zhang, X. Nanofibrous Kevlar Aerogel Threads for Thermal Insulation in Harsh Environments. *ACS Nano* **2019**, *13*, 5703–5711.

(4) Huang, J.; Li, J.; Xu, X.; Hua, L.; Lu, Z. *In Situ* Loading of Polypyrrole onto Aramid Nanofiber and Carbon Nanotube Aerogel Fibers as Physiology and Motion Sensors. *ACS Nano* **2022**, *16*, 8161–8171.

(5) Li, G.; Hong, G.; Dong, D.; Song, W.; Zhang, X. Multiresponsive Graphene-Aerogel-Directed Phase-Change Smart Fibers. *Adv. Mater.* **2018**, *30*, 1801754.

(6) Li, Y.; Zhang, X. Electrically Conductive, Optically Responsive, and Highly Orientated $Ti_3C_2T_x$ MXene Aerogel Fibers. *Adv. Funct. Mater.* **2022**, *32*, 2107767.

(7) Xu, Z.; Zhang, Y.; Li, P.; Gao, C. Strong, Conductive, Lightweight, Neat Graphene Aerogel Fibers with Aligned Pores. *ACS Nano* **2012**, *6*, 7103–7113.

(8) Li, X.; Dong, G.; Liu, Z.; Zhang, X. Polyimide Aerogel Fibers with Superior Flame Resistance, Strength, Hydrophobicity, and Flexibility Made via a Universal Sol-Gel Confined Transition Strategy. *ACS Nano* **2021**, *15*, 4759–4768.

(9) Du, Y.; Zhang, X.; Wang, J.; Liu, Z.; Zhang, K.; Ji, X.; You, Y.; Zhang, X. Reaction-Spun Transparent Silica Aerogel Fibers. *ACS Nano* **2020**, *14*, 11919–11928.

(10) Xue, T.; Zhu, C.; Feng, X.; Wali, Q.; Fan, W.; Liu, T. Polyimide Aerogel Fibers with Controllable Porous Microstructure for Super-Thermal Insulation under Extreme Environments. *Adv.Fiber Mater.* **2022**, *4*, 1118–1128.
(11) Yang, H.; Wang, Z.; Liu, Z.; Cheng, H.; Li, C. Continuous, Strong, Porous Silk Firoin-Based Aerogel Fibers toward Textile Thermal Insulation. *Polymers* **2019**, *11*, 1899.
(12) Liu, Y.; Zhang, Y.; Xiong, X.; Ge, P.; Wu, J.; Sun, J.; Wang, J.; Zhuo, Q.; Qin, C.; Dai, L. Strategies for Preparing Continuous Ultraflexible and Ultrastrong Poly (Vinyl Alcohol) Aerogel Fibers with Excellent Thermal Insulation. *Macromol. Mater. Eng.* **2021**, *306*, 2100399.
(13) Cai, J.; Liu, S.; Feng, J.; Kimura, S.; Wada, M.; Kuga, S.; Zhang, L. Cellulose-Silica Nanocomposite Aerogels by in Situ Formation of Silica in Cellulose Gel. *Angew. Chem.* **2012**, *124*, 2118–2121.
(14) He, H.; Liu, J.; Wang, Y.; Zhao, Y.; Qin, Y.; Zhu, Z.; Yu, Z.; Wang, J. An Ultralight Self-Powered Fire Alarm E-textile Based on Conductive Aerogel Fiber with Repeatable Temperature Monitoring Performance Used in Firefighting Clothing. *ACS Nano* **2022**, *16*, 2953–2967.
(15) Wang, Y.; Cui, Y.; Shao, Z.; Gao, W.; Fan, W.; Liu, T.; Bai, H. Multifunctional Polyimide Aerogel Textile Inspired by Polar Bear Hair for Thermoregulation in Extreme Environments. *Chem. Eng. J.* **2020**, *390*, 124623.
(16) Wang, Z.; Yang, H.; Li, Y.; Zheng, X. Robust Silk Fibroin/Graphene Oxide Aerogel Fiber for Radiative Heating Textiles. *ACS Appl. Mater. Interfaces* **2020**, *12*, 15726–15736.
(17) Liu, Z.; Lyu, J.; Ding, Y.; Bao, Y.; Sheng, Z.; Shi, N.; Zhang, X. Nanoscale Kevlar Liquid Crystal Aerogel Fibers. *ACS Nano* **2022**, *16*, 15237–15248.
(18) Li, J.; Wang, J.; Wang, W.; Zhang, X. Symbiotic Aerogel Fibers Made via In-Situ Gelation of Aramid Nanofibers with Polyamidoxime for Uranium Extraction. *Molecules* **2019**, *24*, 1821.
(19) Chen, Y.; Zhang, C.; Tao, S.; Chai, H.; Xu, D.; Li, X.; Qi, H. High-Performance Smart Cellulose Nanohybrid Aerogel Fibers as a Platform toward Multifunctional Textiles. *Chem. Eng. J.* **2023**, *466*, 143153.
